# Feed-Based Multi-Mycotoxin Occurrence in Smallholder Dairy Farming Systems of South Africa: The Case of Limpopo and Free State

**DOI:** 10.3390/toxins13020166

**Published:** 2021-02-22

**Authors:** Rumbidzai Changwa, Marthe De Boevre, Sarah De Saeger, Patrick Berka Njobeh

**Affiliations:** 1 Department of Biotechnology and Food Technology, Faculty of Science, Doornfontein Campus, University of Johannesburg, P.O. Box 17011, Gauteng 2028, South Africa; naledichangwa@gmail.com (R.C.); sarah.desaeger@ugent.be (S.D.S.); 2 Center of Excellence in Mycotoxicology & Public Health, Department of Bioanalysis, Ghent University, B-9000 Ghent, Belgium

**Keywords:** multi-mycotoxins, UHPLC-MS/MS, smallholder farming, dairy cattle, dairy feed

## Abstract

Mycotoxin contamination of feed does not only cut across food and feed value chains but compromises animal productivity and health, affecting farmers, traders and consumers alike. To aid in the development of a sustainable strategy for mycotoxin control in animal-based food production systems, this study focused on smallholder farming systems where 77 dairy cattle feed samples were collected from 28 smallholder dairy establishments in the Limpopo and Free State provinces of South Africa between 2018 and 2019. Samples were analyzed using a confirmatory UHPLC–MS/MS (Ultra-high performance liquid chromatography-tandem mass spectrometry) method validated for simultaneous detection of 23 mycotoxins in feeds. Overall, mycotoxins assessed were detected across samples with 86% of samples containing at least one mycotoxin above respective decision limits; up to 66% of samples were found to be contaminated with at least three mycotoxins. Findings demonstrated that deoxynivalenol, sterigmatocystin, alternariol and enniatin B were the most common mycotoxins, while low to marginal detection rates were observed for all other mycotoxins with none of the samples containing fusarenon-X, HT-2-toxin and neosolaniol. Isolated cases of deoxynivalenol (maximum: 2385 µg/kg), aflatoxins (AFB_1_ (maximum: 30.2 µg/kg)/AFG_1_ (maximum: 23.1 µg/kg)), and zearalenone (maximum: 1793 µg/kg) in excess of local and European regulatory limits were found. Kruskal–Wallis testing for pairwise comparisons showed commercial feed had significantly higher contamination for deoxynivalenol and its acylated derivatives, ochratoxin A and fumonisins (FB_1_ and FB_2_), whereas forages had significantly higher alternariol; in addition to significantly higher fumonisin B_1_ contamination for Limpopo coupled with significantly higher enniatin B and sterigmatocystin for Free State. Statistically significant Spearman correlations (*p* < 0.01) were also apparent for ratios for deoxynivalenol/fumonisin B_1_ (r_s_ = 0.587) and zearalenone/alternariol methylether (r_s_ = 0.544).

## 1. Introduction

Dairy cattle rearing constitutes an important aspect of agriculture in South Africa (SA). It contributes significantly to socio-economics and food security with livestock rearing systems comprised of commercial and smallholder farmers. Smallholder farming is used in reference to low input-output farming, whereby farmers participate in production at a capacity not as well-developed, capital-intensive and integrated as established larger-scale commercial farmers. This smallholder farming broadly includes both emerging and communal farmers, whereby Muntswu et al. [1] define emerging farmers as beneficiaries of land reform programs with an excess of 15 milking cows and 1 hectare of land producing a minimum of 100 L/day. Communal farmers are those practising agriculture for subsistence on communally owned land administered by traditional authorities, with less of herd size and subsequent yield [2,3]. Smallholder farming has long been recognized by South African policymakers and stakeholders as the vehicle through which the goals of poverty alleviation and rural development can be achieved; hence, several programs and initiatives have been implemented to this effect [3]. However, several years on, the sector reportedly still makes an insignificant contribution to the aggregate industry [4], with relatively few smallholder farmers thriving and the majorities’ success impeded by numerous constraints often hindering progress to commercialization.

As such, of concern to this study are the class of natural feed contaminants collectively referred to as mycotoxins, derived from unwarranted fungal spoilage of feed materials pre- or post-harvest, or during storage due to prevailing climatic conditions, inadequate facilities or technical know-how. Commercially available mycotoxin-based sources common in livestock rearing systems include cereal grains and residues, oilseeds and their by-products alongside other industrial by-products like brewers’ grain, molasses meal, bone meal, and saltlicks [5,6]. Additionally, natural and ensiled forages and grazing pastures also play a part in livestock mycotoxin exposure often with a wide range of Fusario-toxins as common contaminants [7]. These multi-faceted feeding approaches regardless of the scale of production are all indicative of the ever-present mycotoxin risk in livestock production systems.

A growing number of studies have in the recent past laid focus on mycotoxin oc-currences in South African dairy feeds and feed ingredients [8,9,10,11,12], with limited specifics on smallholder farming systems [13], as reported vastly in other African regions [14,15,16,17,18]. Therefore, having identified for the purpose of this study, mycotoxin-related issues as one of the food safety and quality limiting factors impeding smallholder farmers from progressing to commercial agriculture, this study aimed to provide in-depth data, enabling a more accurate and up-to-date evaluation of mycotoxin occurrences in the South African smallholder dairy farming systems, taking Limpopo and Free State provinces into consideration. To aid in the development of sustainable strategy for mycotoxin control in dairy production systems of South Africa, additional focus has to be paid to smallholder farming systems where there is a more insistent need to routinely monitor and investigate regional mycotoxin occurrences and exposure patterns. Mycotoxins of relevance to this study included the key regulated mycotoxins aflatoxins (AFB_1_, AFB_2_, AFG_1_ and AFG_2_), deoxynivalenol (DON) and its acylated derivatives (3-acetyl deoxynivalenol [3-ADON] & 15-acetyl deoxynivalenol [15-ADON]), zearalenone (ZEN), fumonisins (FB_1_, FB_2_ and FB_3_), ochratoxin A (OTA), T-2 toxin (T-2) and HT-2 toxin (HT-2), alongside other mycotoxins [nivalenol (NIV), diacetoxyscirpenol (DAS), fusarenon X (FUS-X), neosolaniol (NEO), alternariol (AOH), alternariol methylether (AME), roquefortine C (ROQ-C), enniatin B (ENN B) and sterigmatocystin (STERIG)]. Data on the natural occurrence of contaminants may be used to aid impending discussions on legislative policies for the dairy feed industry. Overall, this study provides an independent monitoring scheme for mycotoxins and effective quality assurance for mainstream South African dairy production systems as suppliers of animal products into the national food chain.

## 2. Results and Discussion

### 2.1. Method Performance Characteristics

The analytical method employed was developed and validated as a quantitative confirmatory method for use at Ghent University, Belgium, in accordance with the EU Commission Decision, 2002/657/EC [19]. To allow for good extraction efficiency of various mycotoxins assessed and to obtain satisfactory variability in method performance (recovery, linearity and sensitivity) a combination of multiple solvent extractions and clean-up methods (defatting, SPE and MultiSep AflaZon purification) were used. Additionally, de-epoxydeoxynivalenol (DOM) and zearalanone (ZAN), known structural analogues of DON and ZEN, are not found innately in food and feed matrices and are in this method used as internal standards (IS) to compensate for matrix effects and/or possible losses from sample preparation steps.

For purposes of this investigation, performance characteristics for quantification, based on two broad model blank matrices (compound feed and forage) were assessed for linearity, sensitivity, and process efficiency (recovery) of the method and results are presented in Table 1. Matrix matched calibration curves created by plotting responses versus the concentrations of each analyte, fitted by linear regression over specified working ranges showed good linearity with coefficients of determination (R^2^) ranging from 0.932 to 0.998 for compound feed (with the exception of ZEN (0.788]); 0.933 to 0.998 for forages. Data from spikes of blank extracts of respective feed groups were used for the calculation of limits of detection (LOD) and limits of quantification (LOQ) at increasing standard mix concentrations; to assess sensitivity of the adapted method. For compound feed, LODs ranged from 2.0 µg/kg (DAS) to 158.8 µg/kg (FB_1_) with corresponding LOQs ranging from 4.0–317.7 µg/kg for the same compounds (Table 1). Similarly, LODs for forage samples ranged from 0.87 µg/kg (AFG_1_) to 190.3 µg/kg (FB_1_) with corresponding LOQs ranging from 1.7 to 380.6 µg/kg. Apparent recovery (R_A_), which defines process efficiency, strongly varies depending on the matrix/analyte combination. The achieved mean apparent recoveries from matrix-matched samples at the four spike levels per analyte were found to be in the range of 94% (ZEN) to 102% (NIV) in compound feed and 96% (AOH) to 103% (DAS) in forages.

### 2.2. Mycotoxin Occurrence in Dairy Feeds

**Figure 1:**
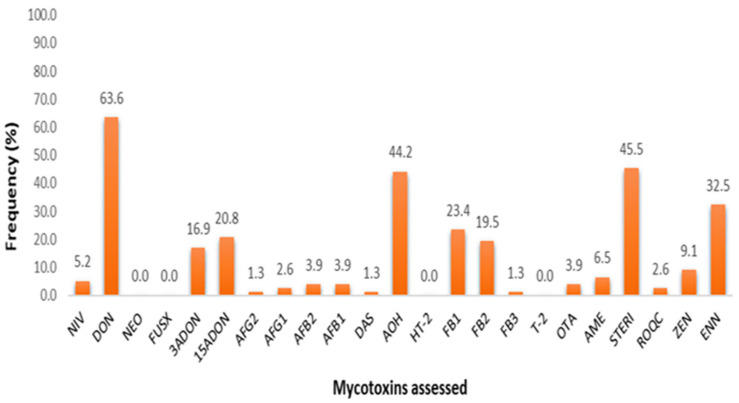
Graphical representation of multi-mycotoxin incidence rates, inclusive of trace amount detections (<CCα).

A total of 77 dairy feeds from smallholder establishments of Limpopo and Free State were tested for multi-mycotoxin contamination using UPLC-MS/MS technology and were found to be contaminated by a range of 2–16 mycotoxins across feed type classifications. Summary statistics across these feed types for means, ranges, numbers and percentages of positive samples are given in Table 2. Total mixed rations (TMR) were found to be the most contaminated feed type with up to 16 mycotoxins found, possibly due to the nature of TMR, with several ingredients (compound and forage) mixed, of-ten to the farmers’ discretion. Additionally, commercially bought compound feed such as the dairy concentrates, meals and pellets were also found to contain 8–9 different mycotoxins. Overall, 20 of the 23 mycotoxins assessed were detected across all feed samples with 86% (66 of 77) of the samples evidenced to have at least one mycotoxin above respective decision limits. FUS-X, HT-2 and NEO were absent across all samples with trace amounts (<CCα) of FB_3_ detected in one sample. Overall incidence rates inclusive of trace amount detections across all mycotoxins are depicted in Figure 1, and associated contamination data is given in Supplementary Materials Table S1.

Findings demonstrate that DON, STERIG, AOH, and ENN B were the most com-monly found mycotoxins with detection rates of 63.6, 45.5, 42.8 and 32.5%, respectively. Indicative of less prevalent patterns were 3-ADON, 15-ADONs, FB_1_, FB_2_ and ZEN (at 23.4–9.1%), while the rest (NIV, AFB_1_, AFB_2_, AFG_1_, AFG_2_, DAS, OTA, AME and ROQ-C) appear to occur marginally across feed samples. In view of mycotoxin detections with concentrations above respective decision limits, additional descriptive statistical analysis of the data on the natural occurrences per farm and by specific feed type presented in Supplementary Materials Tables S3 and S2, respectively.

#### 2.2.1. Regulated Mycotoxins: A South African and European Commission Perspective

Concerning the presence of regulated mycotoxins; these were in this study found in line with the findings of Gruber-Dorninger et al. [20], who reported that DON, FUM, and ZEN were the most prevalent regulated mycotoxins in SA feeds. Where SA regulations [21] for mycotoxins in dairy feedstuff stipulate maximum permissible limits of 5 µg/kg for AFB_1_, 3000 µg/kg for DON, 500 µg/kg for ZEN, 50,000 µg/kg for fumonisins (FUMs), and no specifications for OTA, the EC regulatory limits and guidelines [22] specify a higher 5000 µg/kg for DON, with similar to SA limits for AFB_1_, FUMs, ZEN and an additional 250 µg/kg for OTA; thus, few instances of concentrations in excess of regulatory limits were in our case identified.

As demonstrated in Table 2, even though the *Fusarium* toxin DON showed the highest prevalence of 63.6% at mean and maximum levels of 477.7 and 2385 µg/kg, ZEN, the common DON co-contaminant in animal feed [23], at its much lower prevalence rate of 8.9%, demonstrated a higher mean of 666.7 µg/kg with an upper range of 1793 µg/kg. Despite only two samples (dairy meal and soya bean stover) exceeding the 500 µg/kg SA and EC regulatory limits, general DON and ZEN occurrence patterns appear comparable with [24], whose studies on global feeds across various feed types report the common simultaneous occurrence of DON and ZEN due to similar fungal production lines, but with DON occurring at a higher degree.

Despite differences in intrinsic toxicities [25], the derivatives 3-ADON and 15-ADON, which are also contemplated to be of equivalent toxicity to DON in the animal via their de-acetylation at absorption [26], were found in this study to be less prevalent in overall feed samples occurring more in compound feeds than forages. 3-ADON was found present in 13/77 (16.9%) of overall samples at concentrations ranging from <CCα to 300 µg/kg (mean level: 55.5 µg/kg). Similarly, 15-ADON was detected in 16/77 (20.8%) of all samples at levels ranging 16.0–858.8 µg/kg (mean level 169.6 µg/kg). The highest 3-ADON (300 µg/kg) and 15-ADON (858.8 µg/kg) concentrations detected were from the same maize stover sample that also showed the highest concentration of DON (2385 µg/kg). Of the 49 times DON was detected, the detection ratio for co-occurrence of DON + 3-ADON + 15-ADON: DON + 15-ADON: DON + 3-ADON was 2: 2: 1.25 with DON + 3-ADON + 15-ADON detected in 10% of overall samples.

Regarding ZEN, the previously mentioned low incidence rates appear common-place for South African feed and raw materials with the perception that ZEN maybe a minor yet insistent contaminant in these matrices [10,11,27,28]. The absence of ZEN in grasses, lucerne and silages (Supplementary Materials Table S2) may be attributed to the presence of a less predominantly ZEN producing fungi in these matrices given the specific micro-climates relative to the study. Additionally, as noted by Driehuis [29], proliferation and growth of ZEN producing fungi happen in the field, as the plant grows, thus in the case of grazed grasses and ensilaging forage, which tend to be cut or consumed before full maturity of the plant, it may be rational to assume much lower level contamination in these feeds.

Contrary to the normally reported high rates of fumonisin (FUM) contamination in South African feedstuff [12,24], this study demonstrated lower prevalence rates for FB_1_, FB_2_ and FB_3_ at 23.4, 19.5 and 1.3%, respectively, with accompanying contamination means and maximum values well within the legislated limits (FB_1_—mean level 189.8 µg/kg: maximum level 485.2 µg/kg; FB_2_—mean level 132.4 µg/kg: maximum level 416.9 µg/kg; FB_3_ single detection: <CCα) (data shown in Table 2). The single detection of FB_3_ in trace amounts was from a sample of dairy pellets (Supplementary Materials Table S1). It is clear, though, in comparison to forage material, that compound feeds demonstrated on average much higher FB levels, which appears common for this category of feeds. Additionally, this study reports on a total absence of FUMs in lucerne and molasses meals (Supplementary Materials Table S2), which Knusten et al. [30] assert may be due to the presence of sugar-rich ingredients that may favour the formation of differently structured modified fumonisins from Maillard-type reactions between reducing sugars. Although measurement of these modified fumonisins can ideally be done using indirect approaches, this was not covered in the framework of this work.

Aflatoxins (AFs) and OTA were predominantly absent in most of the samples with marginal prevalence rates of 3.9% each for both total AFs and OTA. Considering the potential risk that AFs pose on human and animal health, this group of mycotoxins is the most commonly monitored and regulated to ensure outbreaks of associated mycotoxicosis are not in question. As infrequent or marginal as these detections may be, they none-the-less contribute to an increased AF intake among lactating dairy cows, which in turn may have carry-over effects of direct proportion in animal tissues and by-products [31,32]. However, contradictory to studies reporting high AF prevalence in SA feeds [9,11,33,34], this study observed much lower detection rates for AFB_1_ (3.9%), AFG_1_ (2.6%); AFB_2_ (3.9%) and AFG_2_ (1.3%) (Table 2). The totality of AF contamination in our study appears to have been from one farm, where the major source might have been a contaminated dairy concentrate used in the formulation of other mixed rations tested. In instances of quantifiable detection, AFB_1_ (mean level: 26.1 µg/kg; maximum level 30.2 µg/kg) and AFG_1_ (mean level: 20.2 µg/kg; maximum level 23.1 µg/kg) independently showed mean levels of contamination exceeding both the SA 10 µg/kg and EU 20 µg/kg regulatory limits for total aflatoxins and the 5 µg/kg SA and EU limit for AFB_1_ in dairy feeds.

Correspondingly, low AF detection rates in SA feeds and feed ingredients have been reported for AFB_1_, AFG_1_, AFB_2_ and AFG_2_ in varying ratios of B/G analogues [10,12,35]. In the current study, total AFs in two of the three positive samples in question were at least 2.5 times above the 20 µg/kg EU regulated limit for total aflatoxins (dairy concentrate: 51.7 µg/kg and total mixed ration 2: 62.9 µg/kg). In the same two samples, the highly toxic AFB_1_ was found at levels up to four times the 5 µg/kg regulatory limit for AFB_1_ alone. Research on AFB_1_ contamination in relation to storage time has demonstrated increases in AFB_1_ levels in compound feeds stored for an excess of one month [36]. Additionally, as with this study, mixed feed rations, when found AF positive, have been reported to be either more frequently or more severely contaminated, this possibly due to their multi-ingredient nature [37]. As with studies such as those of [38], an absence of AFs in forage material is also reported. 

Further ascertaining the assertion that low OTA detections may be the norm in South African dairy feeds over the past years [17,18,20,21], the current study found (Table 2) a 3.9% incidence rate for OTA in the 77 tested samples (mean level: 85.6 µg/kg; maximum level 187.9 µg/kg) with all positives having levels below the 250 µg/kg EC guidance limits specified for cereal-based feeds. While these results are lower than the guidance values, long-term persistent exposure may lead to losses in yield alongside other chronic toxicities in animals [39]. This low prevalence could be accounted for by the complete absence of this mycotoxin in forages as corroborated by other studies [37,38,39,40,41,42], possibly due to most of the OTA producers’ inability to tolerate high acetic acid concentrations characteristic of ensilaging or grass/hay bailing preservative processes [43]. Furthermore, the fact that OTA production largely occurs in species-specific temperatures ranges of 25 to 37 °C and associated lower water activity (below ≈ 0.84) [44], can also account for the comparative absence of this mycotoxin in the Limpopo samples vs. Free State samples. OTA occurrence patterns seem to follow that of AFs, possibly due to the common *Aspergillus* species production lines.

Due to the complexities of matrix effect in both commercial feeds and forages, calibration data were inadequate for the correct quantification of T-2 and HT-2 according to confirmatory criteria specified by the method employed. However, a clear outlier for T-2 contamination at the estimated but unconfirmed concentration of 11 002.6 µg/kg was recorded in a single sample of lucerne, this result was nonetheless disregarded as a confirmed value in the overall results.

#### 2.2.2. Non-Regulated Mycotoxins

The other non-regulated mycotoxins STERIG, AOH, and ENN B were found most frequently at prevalence rates of 45.5, 42.8 and 32.5% at mean concentrations of 25.8 μg/kg (range: <CCα–139.1 μg/kg) 279.2 μg/kg (range: 15.5–3088.2 μg/kg), and 1195.1 μg/kg (range: <CCα–14,230.4 μg/kg), respectively. Enniatins only became an issue of high concern as emerging mycotoxins in recent years, thus information on their occurrence in sub-Saharan Africa (SSA) feedstuff is scarce [28,45]. However, in compliance with our results for the confirmed presence of the most bioactive of the group (ENN B), literature on global feed occurrences is representative of moderate to high detections in compound cereal-based feeds and low to sporadic detections in forages [46,47,48]. To note however would be the high levels found in this study with up to 9 of the 22 detections comparatively in excess of results from Rasmussen and Storm [46] on visibly moldy hotspot silages.

Although data on the adverse health effects of STERIG on dairy cattle are scarce, the AFB_1_-structurally related toxic precursor of AF production is occasionally reported as a contaminant of feeds [40,43,49]. Owing to the shared structural similarities between STERIG and AFs, commonalities in prominent toxicities (hepatotoxicity, genotoxicity and carcinogenicity) remain apparent, with the potency of AFB_1_ considered up to 10 times that of STERIG [43], maximum limits, however, remain unestablished. This study documents the moderate to high-level STERIG contamination with the highest levels found in samples of grass (89.7 μg/kg) and lucerne (139.1 μg/kg) and all three AFB_1_ occurrences were accompanied by STERIG (6.3–30.9 μg/kg). 

With much less known about *Alternaria* mycotoxin occurrences in Southern African feeds, this study documents somewhat comparable prevalence rates of AOH with [12], though at much higher levels (maximum level: 3088.2 μg/kg). Maximum concentrations of AOH were herein found in forage material with highest levels detected in grasses. Monitoring of these possibly mutagenic, genotoxic and precancerous [50] representatives of field spoilage may increasingly be a noteworthy endeavor in SSA given climate change and the insistent need to preserve the farm to fork food chain. Overall, the highest concentrations of AOH, STERIG and ENN B were found in respective samples of grass, lucerne and molasses meal. The remaining mycotoxins, i.e., NIV, DAS, AME and ROQ-C were only found marginally in feed samples. 

### 2.3. Comparisons for Mycotoxin Variability

**Figure 2:**
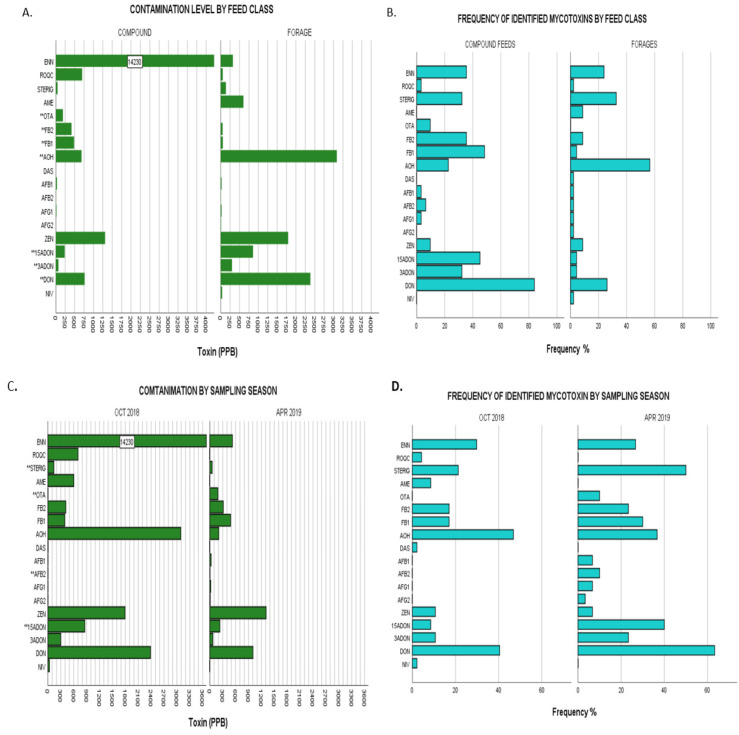
(**A**), Upper limit toxin concentrations for identified mycotoxins in overall dairy feeds from the two different feed classes: compound feeds vs forages. Bars labelled with toxin name followed by a double Asterix (**) showed significant differences of mean ranks by Kruskal–Wallis test at *p* ≤ 0.05. (**B**), Frequencies of identified mycotoxins in same populations by feed class. (**C**), Upper limit toxin concentrations for identified mycotoxins in overall dairy feeds from the two different sampling seasons: October 2018 and April 2019. Bars labelled with toxin name followed by a double Asterix (**) showed significant differences of mean ranks by Kruskal–Wallis test at *p* ≤ 0.05. (**D**), Frequencies of identified mycotoxins in same populations by sampling season. Nivalenol (NIV), deoxynivalenol (DON), 3- acetyl deoxynivalenol (3-ADON), 15- acetyl deoxynivalenol (15-ADONs), zearalenone (ZEN), aflatoxin G_2_ (AFG_2_), aflatoxin G_1_ (AFG_1_), aflatoxin B_2_ (AFB_2_), aflatoxin B_1_ (AFB_1_), diacetoxyscirpenol (DAS), alternariol (AOH), fumonisin FB_1_ (FB_1_,) fumonisin FB_2_ (FB_2_), ochratoxin A (OTA), alternariol monomethyl ether (AME), sterigmatocystin (STERIG), roquefortine C (ROQ-C), enniatin B (ENN B).

**Figure 3:**
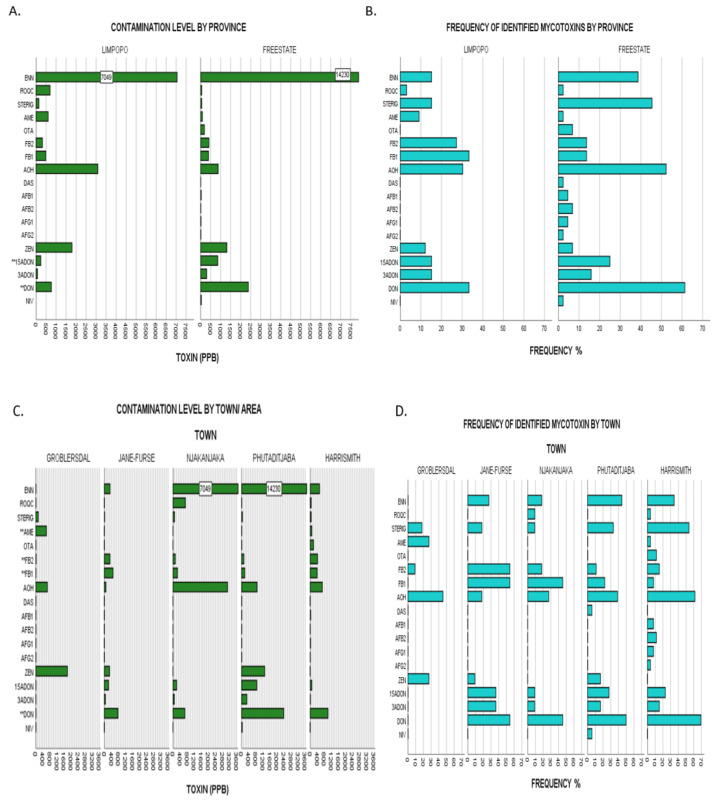
(**A**), Upper limit toxin concentrations for identified mycotoxins in overall dairy feeds from the two different provinces assessed: Limpopo vs Free State. Bars labelled with toxin name followed by a double Asterix (**) showed significant differences of mean ranks by Kruskal–Wallis test at *p* ≤ 0.05. (**B**), Frequencies of identified mycotoxins in same populations by province representative of agro-ecological zone. (**C**), Upper limit toxin concentrations for identified mycotoxins in overall dairy feeds from the five towns assessed: Groblersdal, Jane Furse, Njhakanjhaka, Phutaditjaba and Harrismith. Bars labelled with toxin name followed by a double Asterix (**) showed significant differences of mean ranks by Kruskal–Wallis test at *p* ≤ 0.05. (**D**), Frequencies of identified mycotoxins in same populations by town. Nivalenol (NIV), deoxynivalenol (DON), 3-acetyl deoxynivalenol (3-ADON), 15-acetyl deoxynivalenol (15-ADONs), zearalenone (ZEN), aflatoxin G_2_ (AFG_2_), aflatoxin G_1_ (AFG_1_), aflatoxin B_2_ (AFB_2_), aflatoxin B_1_ (AFB_1_), diacetoxyscirpenol (DAS), alternariol (AOH), fumonisin FB_1_ (FB_1_,) fumonisin FB_2_ (FB_2_), ochratoxin A (OTA), alternariol monomethyl ether (AME), sterigmatocystin (STERIG), roquefortine C (ROQ-C), enniatin B (ENN B).

Taking a further look into contamination profiles by grouping the variables—feed class, sampling season, town, and province (agro-ecological zones) aids in elucidating any mycotoxin issues allowing patterns and possible links to be made for correlational studies with perception–behavioural analysis and exposure analysis for the purpose of regulation and monitoring. Because normality assumptions of parametric tests were not met for all assays, Kruskal–Wallis tests with normal approximation for k independent samples were used to evaluate possible differences in the mean (mean rank) mycotoxin levels across grouping variables. Statistical differences between groups of data were inferred at a significant level of α = 0.05. If significant differences between the groups were found, a Dunn post-hoc test at α = 0.05 with Bonferroni adjustment was used to evaluate the possible differences between incidences of each mycotoxin contamination within the different categories taking into account category means inclusive of all negative samples. Mycotoxin occurrence and distribution in animal feeds is influenced by a wide array of factors which include the nature of feeds (compound or forage), ingredient crop species (type and provenance), regional environmental conditions, season, farm management, amongst others. The effect of feed type and sampling season on mycotoxin detection (frequency and level) in feed samples investigated is presented in Figure 2 and Appendix A: Table A1, while variability (frequency and level) by province and by town is presented in Figure 3 and Appendix A: Table A2.

#### 2.3.1. Comparison by Feed Class 

The proportion of variability in the ranked dependent variables from Kruskal–Wallis nonparametric testing on the effect of feed classes showed increasingly strong significant differences for the levels of OTA < AOH < 3-ADON; FB_2_ < 15-ADON < DON < FB_1_ as denoted by exact p-values given (Appendix A: Table A1). For instance, with regards to FB_1_, the Kruskal–Wallis test showed the strongest statistical significance for differences in FB_1_ levels between the different feed classes (χ^2^(1) = 22.028, *p* = 0.000003) with mean rank FB_1_ levels higher in compound commercial feeds (49.58) compared to forages (31.87) demonstrating higher contamination levels in compound feeds. OTA, on the other hand, showed the weakest significance of variability between the two categories of feed classes with (χ^2^(1) = 4.570, *p* = 0.033) and commercial feeds showing higher mean OTA ranking (41.23) than that of forages (37.5). Overall, commercial feeds showed significantly higher levels of contamination for DON, 3-ADON, 15-ADON, OTA, FB_1_ and FB_2_ while forages had significantly higher AOH levels as illustrated in Figure 2A,B. The remaining mycotoxins showed no significant differences when compared by feed class.

#### 2.3.2. Comparison by Season Sampled

Significant differences of increasing effect were observed across the studied seasons with regards to contamination by STERIG (χ2(1) = 4.45, *p* = 0.035); both AFB_2_ and OTA (χ^2^(1) = 4.825, *p* = 0.028) and 15-ADON (χ^2^(1) = 9.844, *p* = 0.002) with samples of the April 2019 sampling season showing significantly higher mean rankings compared to those of the October 2018 period. A full representation of results is given in Appendix A: Table A1 and illustrated in Figure 2C,D. All remaining mycotoxins showed no significance in variation for this grouping category.

#### 2.3.3. Comparison by Study Regions/Provinces

Irrespective of the year of sampling, mean FB_1_ levels of contamination were significantly higher for Limpopo samples than Free State samples with χ^2^(1) = 4.83, *p* = 0.028. Full nonparametric testing data is herein represented in Appendix A: Table A2. Furthermore, Free State demonstrated higher contamination levels of increasing statistical significance in the contamination of ENN B (χ^2^(1) = 4.27, *p* = 0.039) and STERIG (χ^2^(1) = 6.34, *p* = 0.012). Although there were no other significant differences between levels of all other mycotoxins, Free state samples appear to have higher incidences for DON (61% vs 33%), 15-ADON (25% vs 15%), OTA (7% vs 0%) and AOH (52% vs 30%) than samples from Limpopo as demonstrated in Figure 3A,B. Although differences in climatic conditions across the two agro-ecological regions can explain the observed distribution patterns, other determining factors may come into play such as individual farmer feed management dissimilarities. While not deemed statistically significant, the results for higher DON contamination in the Free State when compared to that of Limpopo (by both magnitude and frequency) (Figure 3A,B) appear fairly comparable with those of [10,35], whereby the Free State maize grain samples had comparatively higher DON contamination profiles than those of Limpopo, given that maize is the main component ingredient in compound feeds.

#### 2.3.4. Comparison by Town Sampled

Pairwise comparison by the town grouping category pointed out significant differences (Appendix A: Table A2) of increasing variability for DON (χ^2^(4) = 11.208, *p* = 0.024), FB_2_ (χ^2^(4) = 12.221, *p* = 0.016), AME (χ^2^(4) = 13.114, *p* = 0.011) and FB_1_ (χ^2^(4) = 17.118, *p* = 0.002). Follow up pairwise comparisons done across all five towns using the Dunn’s method adjusted for errors by the Bonferroni method revealed where differences lay. With regards to DON content, Harrismith samples (mean ranking 46.62) appeared to have a significantly higher content than those from Groblersdal (mean ranking 20.00). FB_2_ comparison demonstrated significant differences between Jane Furse (mean ranking 53.77) and all three areas of Harrismith (mean rank 37.12), Phutaditjaba (mean rank 35.72) and Groblersdal (mean rank 34.36). This is indicative of highest FB_2_ contamination profiles in Jane Furse followed by those from Harrismith, Phutaditjaba and Groblersdal. Similarly, AME showed significant differences in content for samples from Groblersdal in association with those of all four towns i.e., Phutaditjaba, Harrismith, Njhakanjaka and Jane Furse, with the latter comparison showing the strongest variability (associated data in Appendix A: Table A2). Graphical illustrations of frequencies and contamination levels by town are given in Figure 3C,D. 

### 2.4. Mycotoxin Co-Occurrence 

**Figure 4:**
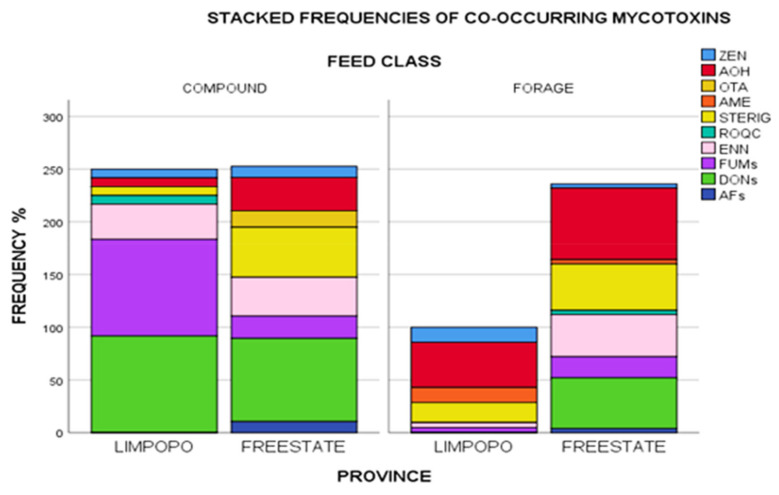
Stacked frequencies (%) of mycotoxins in feeds aggregated by feed class and province. Note: AFs = Sum of AFB_1_, AFB_2_, AFG_1_ and AFG_2_/DONs = Sum of DON, 3ADON, 15 ADON/FUMs = Sum of FB_1_, FB_2_. Zearalenone (ZEN), alternariol (AOH), ochratoxin A (OTA), alternariol monomethyl ether (AME), sterigmatocystin (STERIG), roquefortine C (ROQ-C), enniatin B (ENN B), fumonisins (FUMs), deoxynivalenols (DONs), aflatoxins (AFs).

Realizing, therefore, that toxigenic fungi are at any time capable of producing a variety of mycotoxins, studying single occurrences would give incomplete data for risk assessments. Results of the current study revealed several mycotoxin co-occurrences in a variety of unique combinations given in Supplementary Materials Table S4. A total of twenty-two percent of positive samples were contaminated with one mycotoxin (DON, STERIG, AOH, or ENN B), while 20% of the samples were contaminated with two mycotoxins in various combinations (DON + FB_1_; DON + 15-ADON; DON + AOH; DON + ENN B; AOH + STERIG; AOH + ENN B; ROQ-C + ENN B and STERIG + ENN B). The presence of more than one mycotoxin in over half (66%) of the samples tested underlines the issue of possible toxin interactions and effects these may have on animal health. Taking detection frequencies into account, most commonly occurring mycotoxins included DON and its acylated derivatives (3- and 15- ADONs), fumonisins (FUMs: FB_1_ and FB_2_), AOH, STERIG, ENN B, ZEN and AME in a wide variety of combinations as specified in Supplementary Materials Table S4. Further to this, Figure 4 gives a graphical illustration of proportions of these frequencies aggregated by feed class and province. Compound feeds appear to have variability in co-occurrence with Limpopo samples dominated by DONs + FUMs+ ENN B and lower proportions of ROQ C + STERIG + AOH + ZEN, while Free State compound feeds appear to have been comprised of largely DON and variably lower proportions of STERIG + AOH + ENN B+ FUMs+ OTA + ZEN + AFs. 

Forages from Limpopo, however, had overall lower frequencies of contamination with decreasing proportions of AOH + STERIG + AME + ZEN + FUMs + ENN B co-occurring. Free State forages showed higher frequency proportions with the co-occurrence profile dominated by AOH +DONs + STERIG+ ENN B and lower proportions of FUMS + AFs + ROQ C + AME+ ZEN. Co-contamination by FUMs + ZEN+ AOH + STERIG + ENN B was the only combination present across all grouping categories. The likelihood of simultaneous proliferation of several Fusarium species in feed ingredient plant material could in this instance explain the co-occurrence of FUMs, ZEN, and ENN B. Numerous inferences into species related additive, synergistic and antagonistic effects from co-occurrent combinations have widely been reported in literature [37,38,51], with concurrent occurrences of relevance even at significantly low levels, and exposure often meaning higher metabolic burden on the animal [39].

**Figure A1:**
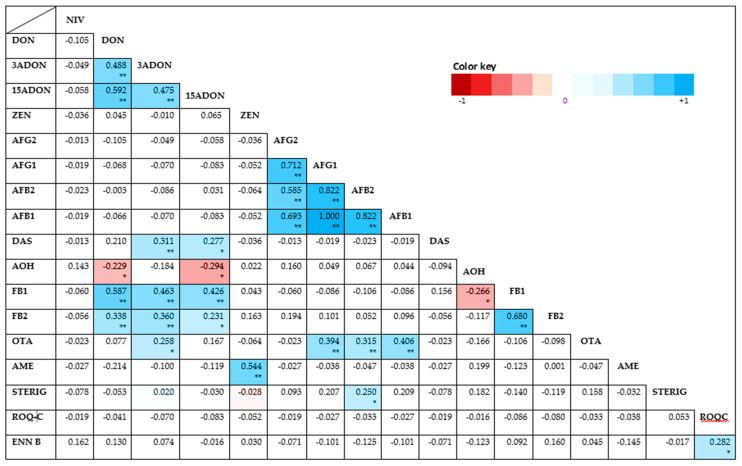
Heat map based on the pairwise Spearman correlation coefficients (rho) between the measured mycotoxin concentrations overall feeds assessed. Darker hues of red indicate a stronger negative correlation while darker hues of blue indicate stronger positive correlations. Unshaded correlations were not significant. ** Correlation is significant at the 0.01 level (2-tailed bivariate). * Correlation is significant at the 0.05 level (2-tailed bivariate).

The Spearman’s rank correlation test on this study revealed significant correlations between the investigated mycotoxins as depicted by the heat map presented in Appendix A: Figure A1. Statistically significant (*p* < 0.01), positive correlations of varying strength were found for several combinations. Strong positive correlations were found for DON: FB_1_ (r_s_ = 0.587, *p* = 0.00000002) and ZEN: AME (r_s_ = 0.544, *p* = 0.0000003). Moderate positive correlations were also found between FB_1_ and both 3- and 15-ADONs (at respective r_s_ = 0.463; 0.426 and *p* = 0.000002; 0.0001); FB_2_ and both DON and 3-ADON (at respective r_s_ = 0.338; 0.360 and *p* = 0.003; 0.01); OTA and AFB_1_, AFG_1_ and AFB_2_ (at respective r_s_ = 0.406; 0.394; 0.315 and *p*= 0.0002; 0.0004; 0.05) and DAS: 3-ADON (r_s_ = 0.311, *p* = 0.06). Weak but positive and still statistically significant (*p* < 0.05) correlations were also found with decreasing magnitude for ROQ-C: ENN B, 15-ADON: DAS, 3-ADON: OTA, AFB_2_: STERIG and 15-ADON: FB_2_. Negative correlations that were statistically significant (*p* < 0.05) were additionally found for 15-ADON: AOH (r_s_ = −0.294, *p* < 0.09) and DON: AOH (r_s_ = 0.229, *p* = 0.046). All other correlations were either non-existent or not significant. Inadequate points of comparison for correlations were here noted as occurrences of toxins are largely matrix and geo-climate based.

## 3. Conclusions

The current data obtained in this study gives clear evidence of isolated cases of AF, DON and ZEN contamination in excess of both SA and/or EC regulatory limits. Despite the other mycotoxins showing contamination levels below advisory limits, the high co-occurrences noted herein unequivocally amplify the risk presented by these feeds on animals based on synergistic, additive and/or antagonistic effects each toxin may have on another as widely documented in literature. Additionally, with most studies on dairy cattle feeds in SSA paying attention largely to regulated mycotoxins, the current study also documents occurrences and levels of contamination on the rarely reported *Alternaria* mycotoxins (AOH and AME) alongside emerging mycotoxins (ENN B, STERIG and ROQ-C) in both cereal- and forage-based feeds. Such regional feed-based data on emerging mycotoxins is scarce; thus, further surveys are needed to build on baseline databases worthwhile for adequate risk assessment. Further to this, noteworthy changes in occurrence patterns were also herein observed with shifts one may attribute to micro-climatic seasonal changes, such as the drought experienced in Limpopo and excessive Free State rainfall during the sampling timeframes, which may have a great impact on overall South African toxigenic fungal patterns. As such, it is our opinion that due to the totality of potential risks mycotoxins pose, regular focus on the trendy “mycotoxin occurrences” in different food/feed systems and different geo-climates remain relevant and highly recommended to achieve higher-level up-to-date databases, which aid toxicological studies towards mycotoxin control. Therefore, this study reports on multiple mycotoxin contamination and co-occurrence issues in smallholder dairy farming systems of Limpopo and Free State, South Africa, in a bid to ensure passable animal health to aid in the safeguarding public health.

## 4. Materials and Methods 

### 4.1. Study Areas and Selection Criteria

Study areas relevant to this research are the two agro-ecologically different provinces of South Africa: Limpopo and Free State. Limpopo province lies in the most northerly part of the country and is characterized by warmer arid to semiarid or sub-humid tropical climates, whereas the centrally located Free State is mainly characterized by subtropical, cooler arid to semiarid climates. Registered active smallholder/emerging dairy milk producers benefitting from developmental projects of the Agricultural Research Council (ARC) within Vhembe and Sekhukhune districts (Limpopo) and Phutaditjaba district (Free State) were therefore selected as a “fit for purpose” population study. Selection of provinces was based on differences in agricultural potential while also taking into account the availability of sampling resources. A total of twenty-eight smallholder dairy farms from different locations (Supplementary Materials Figure S1) thus participated in the study.

A total of 77 dairy cattle feeds were collected from the participating smallholder farms during the months of October to November 2018 and March to April 2019 to (1) cater for feed shortages due to the then prevalent drought conditions in Limpopo and (2) ensure seasonal variation. Samples consisted of commercial feeds and forages namely, dairy concentrates (n = 3), dairy meal (n = 4), pellets (n = 12), molasses (n = 2), ramilick (n = 1), total mixed rations (n = 18), maize stover (n = 3), silages (n = 6), grasses/hay (n = 11), lucerne (n = 12) and soybean stover (n = 5). Sampling points were the individual participating farm storehouses, with one or more representative ingredient batches (bags, bales, pits, heaps) selected at random and one aggregate sample collected as a pooling of several manually collected incremental samples from upper, middle and lower regions of the batch. Resultant aggregate 300–600 g of feed samples were collected into sterile, airtight zip-lock bags, kept chilled and transported to the Food Technology Laboratory, University of Johannesburg, where they were ground/milled to fine particles for homogeneity of the entire aggregate sample, subsampled into smaller representatives (25 g) of each aggregate sample and kept frozen at −18 °C until analysis. Subsamples were subsequently transported to the Center of Excellence in Mycotoxicology and Public Health, Ghent University, Belgium for multi-mycotoxin analysis. For analytical purposes, based on physical appearance and constitution of samples, they were divided into two broadly appropriate matrix classes: compound feeds (dairy concentrates, dairy meals, pellets, molasses meal, ramilick and 9 total mixed rations) and forages (maize stover, grasses/hay, silages, 9 total mixed rations, lucerne and soybean stover).

### 4.2. Mycotoxin Analysis

#### 4.2.1. Chemicals and Reagents

All water used in the preparation of solutions was obtained from a Milli-Q reagent water system (Millipore Corp., Brussels, Belgium). Solvents comprising of high performance liquid chromatograpy (HPLC) grade acetonitrile (Biosolve, Valkenswaard, The Netherlands), and acetic acid (Merck, Leuven, Belgium) were used for extract preparations. N-hexane HiperSolv Chromanorum (VWR International, Leuven, Belgium) was used for defatting. Aqueous and organic mobile phases were made from LC-MS grade absolute methanol (Biosolve, Valkenswaard, Netherlands), UPLC-MS grade acetic acid (Biosolve, Valkenswaard, The Netherlands) and ammonium acetate (Merck, Darmstadt, Germany). Disolol/ethanol (Chemlab, Zedelgem, Belgium) was used for cleaning the apparatus and equipment between samples.

Individual certified mycotoxin solid standards of zearalanone (ZAN) internal standard (IS), alternariol and alternariol monomethylether were purchased from Sigma-Aldrich, (Overijse, Belgium). De-epoxydeoxynivalenol (DOM: IS), aflatoxin mix (AFB_1_, AFB_2_, AFG_1_, and AFG_2_), diacetoxyscirpenol, deoxynivalenol, 3- and 15- acetyl- deoxynivalenol, fumonisin mix (FB_1_, FB_2_), fusarenon-X, HT-2 toxin, T-2 toxin, nivalenol, neosolaniol, ochratoxin A, sterigmatocystin, and zearalenone were obtained as certified mycotoxin standard solutions in acetonitrile from Biopure (Romerlabs, Oostvoorne, Netherlands). Fumonisin B_3_ was obtained from the South African Medical Research Council (Tygerberg, South Africa), enniatin B from Fermentek (Jerusalem, Israel), and roquefortine-C from Alexis Biochemicals (Enzo Life Sciences, Belgium). Stock solutions (1 mg/mL) were prepared from solid mycotoxin standards by dissolving supplied powders with 1000 μL of methanol per mg of standard; with the exceptions of AOH and AME reference components which were dissolved in 40/60 (*v*/*v*) dimethylformamide/methanol. Further dilution of stock solutions was done accordingly to attain specific work solution concentrations. In accordance with the presence or lack of European Commission maximum limits and or guidance values for mycotoxins in animal feeds, two different standard mixtures were used for addition to the spikes, the “legislation” and “not in legislation” mixes. Standard mixes reconstituted in methanol contained:

1. Legislation mix: AFB_1_, AFB_2_, AFG_1_ and AFG_2_ at concentrations of ±2 ng/µL each, OTA at ±5 ng/µL, ZEN, HT-2 and T-2 at ±10 ng/µL each and DON, FB_1_, FB_2_ at ±40 ng/µL each.
2. Not in legislation mix: NIV, FUS-X and AME at individual concentrations of ±20 ng/µL; NEO and AOH at ±10 ng/µL each; 3-ADON and STERIG at ±5 ng/µL each; alongside FB_3_, DAS, ROQ-C and 15-ADON at concentrations of ±25 ng/µL, ±0.5 ng/µL, ±1 ng/µL and ±2.5 ng/µL, respectively.

All standard mixes and working solutions were deemed viable with storage periods of up to 6 months (within expiration period of stock solutions) at −18 °C. 

#### 4.2.2. Sample Preparation, Extraction and Clean-Up 

Sample preparation for the quantitative LC-MS/MS analysis was done in accordance to a quantitative method validated for the determination of mycotoxins in animal feeds as described by Monbaliu et al. [41]. Briefly, 5 g of all unknown samples and blank samples were weighed into 50 mL falcon tubes for extraction. Prior to each batch extraction, internal standards of ZAN (10 ng/μL) and DOM (50 ng/μL) were added at respective volumes of 100 μL and 25 μL to each of the unknown samples, one blank and five spikes. Samples designated spikes were further spiked with known concentrations of mycotoxin mixtures 1 and 2 (Section 4.2.1) and ENN B at four different concentration levels: 0.5, 1, 1.5 and 1.5 times the cut-off (CO) for preparation of the calibration curves. Cut-off levels are in this method used to cater for the absence of minimum required performance limits (MRPLs) for mycotoxins in feeds as detailed by Monbaliu et al. [41].

Samples were left in the dark for 15 min at room temperature then extracted using 20 mL of extraction solvent: acetonitrile/water/acetic acid (79: 20: 1, *v*/*v*/*v*) via agitation on an overhead shaker AG6A (Exacta, Mery-sur-Oise, France) for 1 hour followed by centrifugation at 3300 g for 15 min. Subsequent purification of individual supernatants was achieved on C18-E Strata solid phase extraction (SPE) columns (Phenomenex, Utrecht, The Netherlands) pre-conditioned with extraction solvent and mounted on vacuum elution manifolds. Eluents were collected under gravity into 25 mL volumetric flasks, taking care not to dry the SPE columns. Extraction and purification steps were repeated using 5 mL of the extraction solvent which was again purified into the same coded volumetric flasks and SPE columns dried under vacuum. Subsequent volumes obtained per sample were adjusted and made up to the 25 mL mark using the extraction solvent. Resultant extracts were defatted by addition, agitation, centrifugation and removal of 10 mL n-hexane. Defatted extracts were aliquoted into two for further purification using two different modes of clean-up. Aliquots of 10 mL of the defatted extracts were for the first clean-up method filtered through Whatman glass-microfilters (VWR International, Leuven, Belgium). For the second method, aliquots of 10 mL of the defatted extract were thoroughly mixed and acidified with 20 mL of acetonitrile/acetic acid (99: 1 *v*/*v*), and the resultant 30 mL passed through MultiSep 226 AflaZon+ columns (Romer Labs, Oostvoorne, The Netherlands) into 50 mL falcon tubes with additional 5 mL acetonitrile/acetic acid (99/1, *v*/*v*) washing of the columns. Thereafter, 2 mL of the filtered extract from the first clean-up was combined with the MultiSep 226 eluate and evaporated to dryness under gentle nitrogen flow. Resultant residues were reconstituted in 150 µL of injection mobile phase with vortex and centrifugation for 5 min at 10,000× *g* in 0.22 μm PVDF Durapore centrifugal filters (Merck Millipore, Molsheim, France). Resultant filtrates were transferred into HPLC vials with micro inserts and readied for LC-MS/MS analysis.

#### 4.2.3. UHPLC–MS/MS Analysis

Chromatographic separation, detection and quantification was performed on a Waters Acquity UPLC system (Waters, Zellik, Belgium) coupled to a Quattro Premier XE triple quadrupole mass spectrometer (Waters, Zellik, Belgium). The chromatographic system was equipped with a Symmetry C18 column (5 μm, 150 × 2.1 mm) and a Symmetry C18 guard column (3.5 μm, 10 × 2.1 mm), both from the same supplier (Waters, Zellik, Belgium). Exactly 10 μL of sample extracts were injected into the system with the column oven temperature kept at room temperature (25 °C). A gradient elution program (Supplementary Materials Table S5) was followed using an aqueous mobile phase A (water/methanol/acetic acid [94: 5: 1, *v*/*v*/*v*] and 5 mM ammonium acetate) alongside an organic mobile phase B (water/methanol/acetic acid [2: 97: 1, *v*/*v*/*v*] and 5 mM ammonium acetate) run at a flow rate of 0.3 mL/min and sample run time of 30 min.

The triple quadrupole mass spectrometer was operated in positive electrospray ionization (ESI+) mode with ESI source and desolvation temperatures set at 120 and 400 °C, respectively. A capillary voltage set of 20 kV was used. Nitrogen was used as the spray gas with cone and desolvation gas flows maintained at 50 and 800 L/h, respectively. Data acquisition was performed in multiple reaction monitoring (MRM) mode. The analytical method simultaneously investigated the presence of 23 mycotoxins in feed samples. For each analyte, a precursor ion and cone voltage at which ions were most abundant were determined, and further to this, at least two fragment ions were selected alongside their respective collision energies. Selected MRM transitions and associated MS conditions used are given in Supplementary Materials Table S6.

All instrumental data attainment and processing were performed using Mass Lynx™ and Quan Lynx^®^ version 4.1 software (Waters, Manchester, UK). Subsequent data processing and calculations were executed in Microsoft Excel Office 365 and IBM SPSS statistics version 26.

#### 4.2.4. Method Performance 

Accurate quantification was ensured by making use of internal standards for DOM and ZAN. This was achieved by measuring relative response ratios between target mycotoxin analytes and their corresponding IS, thereby allowing for corrections or compensation of any signal variations that may have been caused by matrix effects or sample loss during extraction and/or clean-up. Quantitation was performed using matrix-matched calibration plots constructed by applying the least-squares method and plotting the relative peak area (ratio of response of the mycotoxin to that of the corresponding peak area of IS) against the spiked concentrations. Since multi-mycotoxin-clean samples are unlikely to come by in feed matrices, quantitative calibratory deductions on Quan Lynx software are based on least contaminated samples deemed “blanks”. Thus, in the event that an assessed “blank” is found to be contaminated, modifications catering for this are made with correction factors calculated accordingly and applied.

Validation studies for the employed method caters for the ambiguity of LODs and false positives/negatives by the establishment of a decision limit (CCα) [19]. This decision limit was defined as the concentration corresponding to the y-intercept plus 1.64 times the residual standard deviation of the intercept for substances without established maximum limits. In the case of substances like AFB_1_ with set maximum limits, CCα was defined as the corresponding concentration at the maximum limit plus 1.64 times the standard deviation within standard deviation of the laboratory precision. Results above the method CCα values per toxin were therefore interpreted to contain the analyte with good probability. The linearity was assessed for each mycotoxin in standard solution and feed matrix by evaluating their linear regression models and evaluating the lack-of-fitness test. Method sensitivity was estimated from the LOD; calculated as three times the ratio of the residual standard error of the intercept and the slope of the standard linear curve as in Equation (1). Similarly, the limit of quantification (LOQ) was six times the aforementioned ratio; Equation (2).

(1) $$\mathrm{LOD}=3\times \left(\mathrm{SDres}÷\mathrm{slope}\right)$$

(2) $$\mathrm{LOQ}=6\times \left(\mathrm{SDres}÷\mathrm{slope}\right)$$

Additionally, the calculated limit values were supported by signal-to-noise ratios (S/N), which according to IUPAC guidelines should be > 3 (LOD) and > 10 (LOQ) for both fragment ions on chromatograms of the lowest spikes. Apparent recoveries were determined using matrix-matched calibration plots and calculated according to IUPAC as the percentage of the ratio of detected concentration values from matrix-matched calibration curves of blanks to their respective theoretical spiking concentrations [42]. Additional to note would be that the employed method takes into account four prerequisite identification criteria that must be simultaneously fulfilled in considering positive results according to [19]:

1. A minimum of two selected fragment ions (three identification points, where one fragment ion = 1.5 points).
2. A signal-to-noise ratio >3 for both fragments.
3. A relative retention time of ± 2.5% with regards to the IS.
4. The ratio of the relative intensity of ions and spikes of similar concentrations must be comparable and range within acceptable limits (deduction not shown). Relative intensity is expressed as a percentage of intensity of the most abundant ion.

## Figures and Tables

**Table 1:** Method performance characteristics of the adapted method based on two model blank matrices; compound feeds and forages, for all studied mycotoxins.

			Compound Feeds					Forage				
**Mycotoxin**	**Calibration Range** **(µg/kg)**	Method Validated CCα (µg/kg)	Calibration Equation Y=	R^2^	Apparent Recovery (Mean ± SD) (%)	LOD (µg/kg)	LOQ (µg/kg)	Calibration Equation Y=	R^2^	Apparent Recovery (Mean ± SD) (%)	LOD (µg/kg)	LOQ (µg/kg)
NIV	137.5–550	36.22	0.00071x + 0.02352	0.983	101.57 ± 10.30	70.4	140.8	0.00074x + 0.00137	0.978	101.12 ± 11.32	67.5	135.0
DON	200–800	60.61	0.004705x − 0.04196	0.994	99.99 ± 3.80	58.9	117.7	0.005306x − 0.1856	0.960	99.94 ± 10.01	116.4	232.7
NEO	62.5–250	8.60	0.007308x + 0.1189	0.994	100.89 ± 5.49	19.4	38.9	0.01092x − 0.07249	0.979	101.16 ± 11.23	36.9	73.9
FUS-X	100–400	16.58	0.002074x + 0.06591	0.990	101.18 ± 8.37	38.4	76.8	0.002034x + 0.1797	0.961	101.23 ± 14.24	66.2	132.3
3-ADON	25–100	4.90	0.002594x − 0.01132	0.993	98.98 ± 6.79	8.2	16.4	0.00288x + 0.06729	0.982	101.66 ± 11.57	18.9	37.7
15-ADON	12.5–50	3.07	0.002643x + 0.02861	0.985	100.02 ± 5.95	6.04	12.0	0.00689x − 0.01571	0.994	99.08 ± 5.97	2.3	4.7
AFG_2_	12.5–50	2.39	0.03527x + 0.07185	0.989	99.47 ± 7.06	5.1	10.2	0.04676x − 0.02576	0.965	100.08 ± 9.97	6.9	13.7
AFG_1_	10–40	1.93	0.0486x − 0.01739	0.997	99.67 ± 4.14	2.1	4.2	0.06081x + 0.01183	0.995	99.71 ± 3.09	0.9	1.7
AFB_2_	7.5–30	1.53	0.05322x + 0.04908	0.989	99.42 ± 7.44	3.0	6.0	0.05568x + 0.01857	0.958	99.78 ± 9.63	4.2	8.4
AFB_1_	10–40	6.75	0.04460x + 0.04191	0.987	99.21 ± 8.59	4.4	8.8	0.07617x − 0.36928	0.998	99.96 ± 2.26	4.5	9.0
DAS	2.5–10	0.67	0.02847x − 0.002919	0.956	101.50 ± 10.25	2.0	4.0	0.04995x − 0.07476	0.944	103.42 ± 18.42	3.1	6.1
AOH	50–200	11.98	0.003834x + 0.09500	0.998	100.39 ± 2.66	8.8	17.6	0.01428x + 0.56141	0.913	96.40 ± 16.46	35.5	71.1
HT2	100–400	9.23	0.000166x − 0.003306	0.971	99.46 ± 10.28	66.9	133.9	-	-	-	-	-
FB_1_	250–1000	31.84	0.005176x + 0.37395	0.974	98.27 ± 13.88	158.8	317.7	0.005004x − 0.256436	0.943	100.20 ± 12.80	190.3	380.6
T2	100–400	9.38	0.000353x + 0.01336	0.978	98.58 ± 12.25	57.7	115.4	-	-	-	-	-
FB_3_	150–600	23.18	0.008669x+ 0.66442	0.994	99.72 ± 3.40	44.6	89.2	0.01194x + 0.173292	0.988	100.74 ± 8.05	65.2	130.4
OTA	37.5–150	3.44	0.004142x − 0.00402	0.980	98.42 ± 12.25	20.9	41.9	0.00903x + 0.020303	0.993	99.84 ± 3.88	5.8	11.7
FB_2_	175–700	24.37	0.00489x + 0.07337	0.989	98.78 ± 7.92	72.7	145.3	0.01021x − 0.34471	0.977	100.01 ± 7.63	71.2	142.6
AME	100–400	17.75	0.00051x − 0.01590	0.964	98.30 ± 10.95	74.9	149.8	0.002131x + 0.27616	0.930	96.83 ± 23.57	78.3	156.7
STERIG	25–100	4.75	0.01245x + 0.081945	0.989	99.83 ± 4.88	10.4	20.7	0.05295x + 1.5101	0.980	98.36 ± 12.08	14.3	28.6
ROQ-C	5–20	1.08	0.00227x − 0.007229	0.957	101.29 ± 15.52	4.1	8.3	0.02645x + 0.00973	0.942	98.47 ± 20.14	1.0	2.0
ZEN	60–240	17.85	0.000375x + 0.01078	0.788	94.15 ± 17.47	120.7	241.4	0.00223x + 0.17914	0.957	99.57 ± 11.82	70.5	141.0
** ENN B	40–160	-	0.004967x + 0.05773	0.932	99.61 ± 12.65	41.9	83.8	0.05929x − 0.79199	0.933	96.78 ± 21.58	40.4	80.7

LEGEND: CCα values adapted as a method parameter for confirmatory analysis. Calibration data selected as the best of two runs for each matrix and R^2^, LOD, LOQ were based on these equations with limit of detection (LOD) calculated as three times the ratio of the residual standard error of the intercept and the slope of the standard linear curve and limit of quantification (LOQ) calculated as six times the aforementioned ratio. ** Semi-quantitative result, -: values not determined. Nivalenol (NIV), deoxynivalenol (DON), neosolaniol (NEO), fusarenon X (FUS-X), 3- acetyl deoxynivalenol (3-ADON), 15- acetyl deoxynivalenol (15-ADONs), aflatoxin G_2_ (AFG_2_), aflatoxin G_1_ (AFG_1_), aflatoxin B_2_ (AFB_2_), aflatoxin B_1_ (AFB_1_), diacetoxyscirpenol (DAS), alternariol (AOH), HT-2 toxin (HT-2), fumonisin FB_1_ (FB_1_,) T-2 toxin (T-2), fumonisin FB_3_ (FB_3_), ochratoxin A (OTA), fumonisin FB_2_ (FB_2_), alternariol monomethylether (AME), sterigmatocystin (STERIG), roquefortine C (ROQ-C), zearalenone (ZEN),and enniatin B (ENN B).

**Table 2:** Summary statistics for all positive detections across specific feed classifications (compound feeds vs forages) and overall feeds.

	Compound Feeds (N = 31)				Forages (N = 46)				Total (N = 77)			
	N (pos)	Concentration (µg/kg)			n (pos)	Concentration (µg/kg)			n (%) pos	Concentration (µg/kg)		
		Min	Max	Mean		Min	Max	Mean		Min	Max	Mean
NIV	0	nd	nd	nd	4	<CCα	36.9	36.9	4 (5.2)	<CCα	36.9	36.9
DON	27	<CCα	766.2	412.9	22	<CCα	2385.4	618.3	49 (63.6)	<CCα	2385.4	477.7
3ADON	10	8.4	70.1	32.8	3	<CCα	300.0	169.2	13 (16.9)	<CCα	300.0	55.5
15ADON	14	16.0	235.5	122.6	2	138.6	858.8	498.7	16 (20.8)	16.0	858.8	169.6
ZEN	3	293.9	1303.9	636.1	4	96.7	1793.7	688.5	7 (9.1)	96.7	1793.7	666.0
AFG_2_	0	nd	nd	nd	1	11.1	11.1	11.1	1 (1.3)	11.1	11.1	11.1
AFG_1_	1	17.2	17.2	17.2	1	23.1	23.1	23.1	2 (2.6)	17.2	23.1	20.2
AFB_2_	2	2.2	4.3	3.2	1	6.8	6.8	6.8	3 (3.9)	2.2	6.8	4.4
AFB_1_	2	<CCα	30.2	30.2	1	21.9	21.9	21.9	3 (3.9)	<CCα	30.2	26.1
DAS	0	nd	nd	nd	1	3.4	3.4	3.4	1 (1.3)	3.4	3.4	3.4
AOH	7	17.9	683.4	181.3	26	15.5	3088.2	305.6	33 (42.8)	15.5	3088.2	279.2
FB_1_	15	71.3	485.2	208.9	3	<CCα	55.4	47.4	18 (23.4)	<CCα	485.2	189.8
FB_2_	11	66.4	416.2	168.8	4	21.1	50.5	32.3	15 (19.5)	21.1	416.9	132.4
FB_3_	1	<CCα	<CCα	<CCα	0	nd	nd	nd	1 (1.3)	<CCα	<CCα	-
OTA	3	19.4	187.9	85.6	0	nd	nd	nd	3 (3.9)	19.4	187.9	85.6
AME	0	nd	nd	nd	5	<CCα	603.2	229.2	5 (6.5)	<CCα	603.2	229.2
STERIG	10	6.8	46.1	17.4	25	<CCα	139.1	31.6	35 (45.5)	<CCα	139.1	25.8
ROQC	1	699.9	699.9	699.9	1	54.4	54.4	54.4	2 (2.6)	54.4	699.9	377.2
** ENN	13	<CCα	14230.4	2296.5	12	<CCα	225.2	93.7	25 (32.5)	<CCα	14230.4	1143.1

LEGEND: Calculation of mean and range values was based on positive samples. N (pos)**:** number of positive samples, whereby values above decision limit (<CCα) are said to contain analyte with good probability. ** semi-quantitative result, nd: not detected, <CCα: trace amount detection as established by decision limit. Nivalenol (NIV), deoxynivalenol (DON), 3- acetyl deoxynivalenol (3-ADON), 15- acetyl deoxynivalenol (15-ADONs), zearalenone (ZEN), aflatoxin G_2_ (AFG_2_), aflatoxin G_1_ (AFG_1_), aflatoxin B_2_ (AFB_2_), aflatoxin B_1_ (AFB_1_), diacetoxyscirpenol (DAS), alternariol (AOH), fumonisin FB_1_ (FB_1_,) fumonisin FB_2_ (FB_2_), fumonisin FB_3_ (FB_3_), ochratoxin A (OTA), alternariol monomethylether (AME), sterigmatocystin (STERIG), roquefortine C (ROQ-C) and enniatin B (ENN B).

## Data Availability

The data presented in this study are available on request from the corresponding authors.

## Supplementary Materials

The following are available online at https://www.mdpi.com/2072-6651/13/2/166/s1, Table S1: Concentrations (μg/kg) of the detected toxins (unadjusted data). Table S2: Summary statistics for all positive detections across specific feed types along with overall feeds. Table S3: Overview of prevalence data of investigated mycotoxins in smallholder dairy feeds specifying individual farm means concentrations alongside overall contamination data (N = 77). Table S4: Mycotoxin co-occurrence combinations found in study. Table S5: LC Gradient program employed., Table S6: MS/MS parameters for determination of 23 mycotoxins and 2 internal standards. Figure S1. Map of South Africa showing towns and smallholder farming areas of interest to this study.

## Appendix A

**Table A1:** Pairwise comparison for variability by feed class and by season (N = 77).

		Grouping Variable: Feed Class					Grouping Variable: Season				
	Overall Mean ^a^ (µg/kg)	Group (n)	Mean ^a^ (µg/kg)	Kruskal–Wallis H χ^2^	Asymptotic. Sig. *p*	Mean Rank	Group (n)	Mean ^a^ (µg/kg)	Kruskal–Wallis H χ^2^	Asymptotic. Sig. *p*	Mean Rank
NIV	0.48	Comp (31)	nd	0.674	0.412	-	O2018 (47)	0.79	0.638	0.424	-
		For (46)	0.80				A2019 (30)	nd			
DON	235.77	Comp (31)	346.29 ^a^	20.037	0.000008	51.97	O2018 (47)	207.88	3.25	0.071	-
		For (46)	161.29 ^b^			30.26	A2019 (30)	279.47			
3-ADON	8.65	Comp (31)	10.56 ^a^	10.085	0.001	45.23	O2018 (47)	9.17	2.12	0.145	-
		For (46)	7.35 ^b^			34.8	A2019 (30)	7.82			
15-ADON	35.24	Comp (31)	55.36 ^a^	17.304	0.000032	48.16	O2018 (47)	27.07 ^b^	9.844	0.002	34.47
		For (46)	21.68_b_			32.83	A2019 (30)	48.05 ^a^			46.1
ZEN	60.55	Comp (31)	61.56	0.016	0.901	-	O2018 (47)	65.07	0.344	0.558	-
		For (46)	59.86				A2019 (30)	53.46			
AFG_2_	0.14	Comp (31)	nd	0.67	0.412	-	O2018 (47)	nd	1.567	0.211	-
		For (46)	0.24				A2019 (30)	0.37			
AFG_1_	0.52	Comp (31)	0.55	0.07	0.792	-	O2018 (47)	nd	3.175	0.075	-
		For (46)	0.50				A2019 (30)	1.34			
AFB_2_	0.17	Comp (31)	0.21	0.84	0.361	-	O2018 (47)	nd ^b^	4.825	0.028	37.5
		For (46)	0.15				A2019 (30)	0.44 ^a^			41.35
AFB_1_	0.68	Comp (31)	0.97	0.09	0.763	-	O2018 (47)	nd	3.175	0.075	-
		For (46)	0.48				A2019 (30)	1.74			
DAS	0.04	Comp (31)	nd	0.674	0.412	-	O2018 (47)	0.07	0.638	0.424	-
		For (46)	0.07				A2019 (30)	nd			
AOH	119.68	Comp (31)	40.95 ^b^	8.355	0.004	30.9	O2018 (47)	172.67	1.638	0.201	-
		For (46)	172.73 ^a^			44.46	A2019 (30)	36.65			
FB_1_	41.91	Comp (31)	101.05 ^a^	22.028	0.000003	49.58	O2018 (47)	23.75	2.483	0.115	-
		For (46)	2.06b			31.87	A2019 (30)	70.37			
FB_2_	25.79	Comp (31)	59.89 ^a^	10.24	0.001	45.87	O2018 (47)	19.45	0.653	0.419	-
		For (46)	2.81 ^b^			34.37	A2019 (30)	35.72			
OTA	3.33	Comp (31)	8.28 ^a^	4.570	0.033	41.23	O2018 (47)	nd ^b^	4.825	0.028	37.5
		For (46)	nd ^b^			37.5	A2019 (30)	8.56 ^a^			41.35
AME	11.91	Comp (31)	nd	2.804	0.094	-	O2018 (47)	19.51	2.656	0.103	-
		For (46)	19.93				A2019 (30)	nd			
STERIG	8.37	Comp (31)	5.53	0.072	0.788	-	O2018 (47)	8.05 ^b^	4.45	0.035	35.43
		For (46)	10.29				A2019 (30)	8.87 ^a^			44.6
ROQ-C	9.80	Comp (31)	22.58	0.091	0.763	-	O2018 (47)	16.05	1.293	0.255	-
		For (46)	1.18				A2019 (30)	nd			
ENN B	341.45	Comp (31)	814.88	2.825	0.093	-	O2018 (47)	531.01	0.317	0.573	-
		For (46)	22.39				A2019 (30)	44.46			

LEGEND: Mean pairs with different superscripts show significant differences at *p* < 0.05 with ^a^ of higher mean rank than ^b^. ^a^ Arithmetic mean of all samples tested; -: none significant result; nd: not detected; n: group number of samples analyzed. Comp: compound feeds/For: forages; O2018: October 2018/A2019: April 2019. Nivalenol (NIV), deoxynivalenol (DON), 3- acetyl deoxynivalenol (3-ADON), 15- acetyl deoxynivalenol (15-ADONs), zearalenone (ZEN), aflatoxin G2 (AFG2), aflatoxin G1 (AFG1), aflatoxin B2 (AFB2), aflatoxin B1 (AFB1), diacetoxyscirpenol (DAS), alternariol (AOH), fumonisin FB1 (FB1,) fumonisin FB2 (FB2), fumonisin FB3 (FB3), ochratoxin A (OTA), alternariol monomethylether (AME), sterigmatocystin (STERIG), roquefortine C (ROQ-C) and enniatin B (ENN B).

**Table A2:** Pairwise comparison for variability by province and by town (N = 77).

	Grouping Variable: Province					Grouping Variable: Town					
	Group (n)	Mean ^A^ (µg/kg)	K-Wallis H χ^2^	Asympt. Sig. *p*	Mean Rank	Group (n)	Mean ^A^ (µg/kg)	K-Wallis H χ^2^	Asympt Sig. *p*	Mean Rank	Sig. Pairwise Comparison (sig ^B^)
NIV	Limp (33) FreeS (44)	Nd 0.84	0.75	0.386	-	Grob (11) Jane-F (11) Njhak (11) Phut (18) Harri (26)	nd nd nd 2.05 nd	3.278	0.512	-	n/a
DON	Limp (33) FreeS (44)	175.82 280.73	2.32	0.128	-	Grobl (11) JaneF (11) Njhak (11) Phut (18) Harri (26)	nd 293.84 233.63 323.43 251.17	11.208	0.024	20.00 44.45 40.00 40.00 43.62	Grobl-Harri (0.017)
3-ADON	Limp (33) FreeS (44)	6.66 10.14	0.00026	0.987	-	Grobl (11) JaneF (11) Njhak (11) Phut (18) Harri (26)	nd 13.61 6.37 21.62 2.18	5.857	0.210	-	n/a
15-ADON	Limp (33) FreeS (44)	29.59 39.48	0.55	0.459	-	Grobl (11) JaneF (11) Njhak (11) Phut (18) Harri (26)	nd 69.62 19.15 78.54 12.44	6.589	0.159	-	n/a
ZEN	Limp (33) FreeS (44)	80.28 45.75	0.60	0.439	-	Grobl (11) JaneF (11) Njhak (11) Phut (18) Harri (26)	214.12 26.72 nd 111.83 nd	9.365	0.053	-	n/a
AFG_2_	Limp (33) FreeS (44)	nd 0.25	0.75	0.386	-	Grobl (11) JaneF (11) Njhak (11) Phut (18) Harri (26)	nd nd nd nd 0.43	1.962	0.743	-	n/a
AFG_1_	Limp (33) FreeS (44)	nd 0.92	1.52	0.218	-	Grobl (11) JaneF (11) Njhak (11) Phut (18) Harri (26)	nd nd nd nd 1.55	3.975	0.409	-	n/a
AFB_2_	Limp (33) FreeS (44)	nd 0.30	2.31	0.129	-	Grobl (11) JaneF (11) Njhak (11) Phut (18) Harri (26)	nd nd nd nd 0.51	6.041	0.196	-	n/a
AFB_1_	Limp (33) FreeS (44)	nd 1.18	1.52	0.218	-	Grobl (11) JaneF (11) Njhak (11) Phut (18) Harri (26)	nd nd nd nd 2.00	3.975	0.409	-	n/a
DAS	Limp (33) FreeS (44)	nd 0.08	0.75	0.386	-	Grobl (11) JaneF (11) Njhak (11) Phut (18) Harri (26)	nd nd nd 0.19 nd	3.278	0.512	-	n/a
AOH	Limp (33) FreeS (44)	170.29 81.71	1.65	0.199	-	Grobl (11) JaneF (11) Njhak (11) Phut (18) Harri (26)	119.28 10.43 381.17 81.41 81.92	5.808	0.214	-	n/a
FB_1_	Limp (33) FreeS (44)	71.63 ^a^ 19.63 ^b^	4.83	0.028	43.70 35.48	Grobl (11) JaneF (11) Njhak (11) Phut (18) Harri (26)	nd 145.08 69.82 21.87 18.07	17.188	0.002	30.50 52.95 47.64 38.22 33.58	Harri-JaneF (0.009) Grobl-JaneF (0.012)
FB2	Limp (33) FreeS (44)	38.21 16.47	2.59	0.108	-	Grobl (11) JaneF (11) Njhak (11) Phut (18) Harri (26)	1.92 93.18 19.53 11.11 20.19	12.221	0.016	34.36 53.77 38.68 35.72 37.12	Phut-JaneF (0.023) Harri-JaneF (0.028) Grobl-JaneF (0.033)
OTA	Limp (33) FreeS (44)	nd 5.83	2.31	0.129	-	Grobl (11) JaneF (11) Njhak (11) Phut (18) Harri (26)	nd nd nd nd 9.87	6.041	0.196	-	n/a
AME	Limp (33) FreeS (44)	25.10 2.02	1.79	0.181	-	Grobl (11) JaneF (11) Njhak (11) Phut (18) Harri (26)	75.29 nd nd nd 3.41	13.114	0.011	47.55 37.00 37.00 37.00 38.46	Phut-Grobl (0.014) Harri-Grobl (0.033) Njhak-Grobl (0.040) JaneF-Grobl (0.040)
STERIG	Limp (33) FreeS (44)	8.08 ^b^ 8.59 ^a^	6.34	0.012	32.83 43.63	Grobl (11) JaneF (11) Njhak (11) Phut (18) Harri (26)	14.14 1.93 8.15 8.34 8.77	7.753	0.101	-	n/a
ROQ C	Limp (33) FreeS (44)	21.21 1.24	0.50	0.823	-	Grobl (11) JaneF (11) Njhak (11) Phut (18) Harri (26)	nd nd 63.63 nd 2.09	3.074	0.546	-	n/a
ENN B	Limp (33) FreeS (44)	241.83 ^b^ 416.16 ^a^	4.27	0.039	34.15 42.64	Grobl (11) JaneF (11) Njhak (11) Phut (18) Harri (26)	nd 42.94 682.55 958.62 40.62	7.742	0.101	-	n/a

LEGEND: Mean pairs with different superscripts show significant differences at *p* < 0.05 with ^a^ of higher mean rank than ^b^; ^A^ Arithmetic mean of all samples tested; n: group number of samples analyzed; nd: not detected; -: none significant result; n/a: no applicable pairwise comparison. ^B^ Significance values have been adjusted by the Bonferroni correction for multiple tests; Limp (Limpopo); FreeS (Free State); Grobl (Groblersdal); JaneF (Jane Furse); Njhak (Njhakanjaka); Phut (Phutaditjaba); Harri (Harrismith). Nivalenol (NIV), deoxynivalenol (DON), 3- acetyl deoxynivalenol (3-ADON), 15- acetyl deoxynivalenol (15-ADONs), zearalenone (ZEN), aflatoxin G2 (AFG2), aflatoxin G1 (AFG1), aflatoxin B2 (AFB2), aflatoxin B1 (AFB1), diacetoxyscirpenol (DAS), alternariol (AOH), fumonisin FB1 (FB1,) fumonisin FB2 (FB2), ochratoxin A (OTA), alternariol monomethyl ether (AME), sterigmatocystin (STERIG), roquefortine C (ROQ-C), enniatin B (ENN).
