# Supplementary material for: Feed-Based Multi-Mycotoxin Occurrence in Smallholder Dairy Farming Systems of South Africa: The Case of Limpopo and Free State
Source: Toxins (Basel). 2021 Feb 22;13(2):166. doi: 10.3390/toxins13020166 (PMC7927053; doi:10.3390/toxins13020166)
Supplement: Supplementary file 1 [file toxins-13-00166-s001.pdf]

toxins

MDPI

Supplementary Materials: Feed-Based Multi-Mycotoxin Occurrence in Smallholder Dairy Farming Systems of South Africa: The Case of Limpopo and Free State

Rumbidzai Changwa, Marthe De Boevre, Sarah De Saeger and Patrick Berka Njobeh

Table S1. Concentrations (µg/kg) of the detected toxins (unadjusted data).

		AFG2	AFG1	AFB2	AFB1	STERIG	NIV	DON	3ADON	15ADON	ZEN	OTA	DAS	T2	HT2	FB1	FB2	FB3	ENN B	NEO	FUS-X	AOH	AME	ROQC
FH255	Dairy concentrate							328.6	17.7	53.1		19.4							527.2					
FH250	Dairy concentrate		17.2	4.3	30.2	30.9		84.5				187.9												
LN114	Ramilick							<CCα											7048.6					699.9*
LJ272	Pellets							766.2	54.3	156.3						485.2	168.7							
LJ270	Pellets							592.7	53.4	179.0						289.4	219.9					30.5		
LJ271	Pellets							559.8		235.5						392.0	315.1		130.7					
LJ121	Pellets							406.7	33.6							71.3	76.3		321.0					
FP147	Dairy meal							490.3	50.9	186.0						176.0	128.5		129.1					
FP264	Molasses meal					46.1													432.4					
LN111	Pellets							344.5								52.3	83.1		459.4					
LJ269	Pellets							503.2		195.0	293.9					217.6	178.6							
FP262	Feed 2 unnamed							477.9		144.2									147.5					
LN275	Pellets							669.8	70.1							156.3	131.7							
FH132	Dairy meal																							
LN276	Pellets							583.5		210.6						247.2			<CCα					
FP141	Molasses meal																		14230.4*					
FH259	Pellets					7.6		390.0	9.2	59.3						77.0								
FP267	Dairy meal					6.8		309.9		86.2	1309.9													
FH125	Concentrate																							
FP144	Dairy meal							317.7			304.6					122.9	71.4		1790.3					
LJ119	Pellets					6.8		403.6	8.4							140.4	66.4	<CCα						
LN117	Pellets							433.3								121.2								
LN277	Pellets							538.8								191.0			<CCα					
FH136	TMR					15.0		192.9								392.9	416.9		44.8			214.8		
FH129	TMR							653.7		88.7														
FH258	TMR 1					10.1		239.8	16.3	16.0		49.4												
FH261	TMR					9.0		258.0	13.6	57.7												89.1		
FH128	TMR					29.5		101.2														127.7		
FH260	TMR 2			2.2	<CCα	9.6		375.9		48.7												17.9		
FH127	TMR							617.9														683.4		
FP265	TMR							94.5														106.0		
FP148	Maize stover						<CCα	2385.4	300.0	858.8			3.4			39.3								

FP140	Maize stover/grain		<CCα	<CCα	<CCα								
FP139	Maize stover		<CCα	<CCα									
LN113	Grass 1										3088.2		
FH254	Grass	55.6		<CCα						22.9	208.5		
LJ123	Grass 1												
FP145	Grass										876.8		
LN116	Grass	89.7									648.4		
FP268	Lucerne	36.7											
LG107	Lucerne	16.5											
LN118	Lucerne												
LJ273	Field lucerne	<CCα								20.6			
FP263	Hay 1										151.4		
LN112	Grass										456.3		
FH256	TMR	11.0		<CCα						32.0		<CCα	
LG102	Soya bean stover2	<CCα		<CCα		464.9			21.1			85.1	
LG106	Soya bean meal/stover												
LJ120	Grass			<CCα									
LG278	Grass	<CCα											
LG110	Soybean stover												
FH134	Silage			76.8						225.2			
FH135	Lucerne/crops	15.0									164.7	88.7	
LG109	Commercial TMR	<CCα				96.7				<CCα	128.9	139.9	
LG105	Hay	<CCα									70.8		
FH253	TMR	9.4		321.4						20.6	32.3		
FH131	Total mixed ration	<CCα		295.0						54.3	16.9		
FP146	Total mixed ration	35.4		585.4	38.3	398.4		<CCα		324.0	95.7		
FH133	Maize silage			89.1					50.5		15.5		
LG103	Grass										653.1		
FP142	Lucerne		36.9	<CCα						88.7	136.6		
LG104	Lucerne	139.1									204.1		
FH251	Lucerne	<CCα									120.9		
LJ122	Lucerne	<CCα									84.2		
LG108	Lucerne	<CCα					11002.6*						
LN115	Lucerne	<CCα											
FH249	Dairy mix furu	4.8		<CCα					25.4		118.2		
LJ274	Grass silage	14.4											
LG101	Soya bean stover 1			<CCα		1793.7					255.1	603.2	
FH126	Silage			409.3							52.8		
FH130	TMR/concentrate	14.2		271.1						81.4	39.4		54.4
FH137	Lucerne/bean stover/silage			820.4						47.7	38.8		

FH257	Silage						1004.9						
FP143	Silage mix					17.4	876.0	138.6					
FH252	TMR	11.1	23.1	6.8	21.9	6.3	<CCα			32.1		189.0	
FP266	Mix- feeding trough					7.7	284.6		55.4			35.5	
FP138	Bean stover										112.8	63.4	

*Unconfirmed positive according to confirmatory criteria, <CCα – below decision limit, TMR: total mixed ration (arbitrary content according to farmer).

Table S2. Summary statistics for all positive detections across specific feed types along with overall feeds.

			NIV	DON	3ADON	15ADON	ZEN	AFG2	AFG1	AFB2	AFB1	DAS	AOH	FB1	FB2	FB3	OTA	AME	STERIG	ROQC	**ENN
**Dairy concentrate (n=3)**	n (pos)		0	2	1	1	0	0	1	1	1	0	0	0	0	0	2	0	1	0	1
	(µg/kg)	min	nd	84.5	17.7	53.1	nd	nd	17.2	4.3	30.2	nd	nd	nd	nd	nd	19.4	nd	30.9	nd	527.2
		max	nd	328.6	17.7	53.1	nd	nd	17.2	4.3	30.2	nd	nd	nd	nd	nd	187.9	nd	30.9	nd	527.2
		mean	nd	206.6	17.7	53.1	nd	nd	17.2	4.3	30.2	nd	nd	nd	nd	nd	103.7	nd	30.9	nd	527.2
**Dairy meal (n=4)**	n (pos)		0	3	1	2	2	0	0	0	0	0	0	2	2	0	0	0	1	0	2
	(µg/kg)	min	nd	309.9	50.9	86.2	304.6	nd	nd	nd	nd	nd	nd	122.9	71.4	nd	nd	nd	6.8	nd	129.1
		max	nd	490.3	50.9	186	1309.9	nd	nd	nd	nd	nd	nd	176	128.5	nd	nd	nd	6.8	nd	1790.3
		mean	nd	372.6	50.9	136.1	807.3	nd	nd	nd	nd	nd	nd	149	100	nd	nd	nd	7	nd	960
**Dairy pellets (n=12)**	n (pos)		0	12	6	6	1	0	0	0	0	0	1	12	8	1	0	0	2	0	5
	(µg/kg)	min	nd	344.5	8.4	59.3	293.9	nd	nd	nd	nd	nd	30.5	52.3	66.4	<CCα	nd	nd	6.8	nd	<CCα
		max	nd	766.2	70.1	235.5	293.9	nd	nd	nd	nd	nd	30.5	485.2	315.1	<CCα	nd	nd	7.6	nd	459.4
		mean	nd	516	38.2	172.6	293.9	nd	nd	nd	nd	nd	30.5	203.4	155	<CCα	nd	nd	7.2	nd	303.7
**Molasses meal (n=2)**	n (pos)		0	0	0	0	0	0	0	0	0	0	0	0	0	0	0	0	1	0	2
	(µg/kg)	min	nd	nd	nd	nd	nd	nd	nd	nd	nd	nd	nd	nd	nd	nd	nd	nd	46.1	nd	432.4
		max	nd	nd	nd	nd	nd	nd	nd	nd	nd	nd	nd	nd	nd	nd	nd	nd	46.1	nd	14230.4
		mean	nd	nd	nd	nd	nd	nd	nd	nd	nd	nd	nd	nd	nd	nd	nd	nd	46.1	nd	7331.4
**Ramilick (n=1)**	n (pos)		0	1	0	0	0	0	0	0	0	0	0	0	0	0	0	0	0	0	1
	(µg/kg)	min	nd	<CCα	nd	nd	nd	nd	nd	nd	nd	nd	nd	nd	nd	nd	nd	nd	nd	nd	7048.6
		max	nd	<CCα	nd	nd	nd	nd	nd	nd	nd	nd	nd	nd	nd	nd	nd	nd	nd	nd	7048.6
		mean	nd	<CCα	nd	nd	nd	nd	nd	nd	nd	nd	nd	nd	nd	nd	nd	nd	nd	nd	7048.6
**Total mixed ration**	n (pos)		0	17	3	5	2	1	1	2	2	0	14	3	3	0	1	2	14	1	8
	(µg/kg)	min	nd	<CCα	13.6	16	96.7	11.1	23.1	2.2	<CCα	nd	16.9	<CCα	25.4	nd	49.4	<CCα	<CCα	54.4	<CCα
		max	nd	653.7	38.3	144.2	398.4	11.1	23.1	6.8	21.9	nd	683.4	392.9	416.9	nd	49.4	139.9	35.4	54.4	324
		mean	nd	340.7	22.7	71.1	247.6	11.1	23.1	4.5	21.9	nd	136.4	224.2	158.1	nd	49.4	139.9	13.5	54.4	100.7

**s (n=18)**																					
**Maize**	n (pos)		3	3	2	1	0	0	0	0	0	1	0	1	0	0	0	0	0	0	0
**stover/**	(µg/kg)	min	<CCα	<CCα	<CCα	858.8	nd	nd	nd	nd	nd	3.4	nd	39.3	nd	nd	nd	nd	nd	nd	nd
**grain**		max	<CCα	2385.4	300	858.8	nd	nd	nd	nd	nd	3.4	nd	39.3	nd	nd	nd	nd	nd	nd	nd
**(n=3)**		mean	<CCα	2385.4	300	858.8	nd	nd	nd	nd	nd	3.4	nd	39.3	nd	nd	nd	nd	nd	nd	nd
	n (pos)		0	2	0	0	0	0	0	0	0	0	8	0	0	0	0	0	4	0	1
**Grasses**	(µg/kg)	min	nd	<CCα	nd	nd	nd	nd	nd	nd	nd	nd	208.5	nd	nd	nd	nd	nd	<CCα	nd	22.86
		max	nd	<CCα	nd	nd	nd	nd	nd	nd	nd	nd	3088.2	nd	nd	nd	nd	nd	89.7	nd	22.9
**(n=11)**		mean	nd	<CCα	nd	nd	nd	nd	nd	nd	nd	nd	988.5	nd	nd	nd	nd	nd	72.7	nd	11.4
**Lucerne**	n (pos)		1	2	0	0	0	0	0	0	0	0	6	0	0	0	0	1	9	0	3
	(µg/kg)	min	36.9	<CCα	nd	nd	nd	nd	nd	nd	nd	nd	38.8	nd	nd	nd	nd	88.7	<CCα	nd	20.6
**(n=12)**		max	36.9	820.4	nd	nd	nd	nd	nd	nd	nd	nd	204.1	nd	nd	nd	nd	88.7	139.1	nd	88.7
		mean	36.9	820.4	nd	nd	nd	nd	nd	nd	nd	nd	124.9	nd	nd	nd	nd	88.7	51.8	nd	52.3
**Soya**	n (pos)		0	2	0	0	2	0	0	0	0	0	2	0	1	0	0	2	1	0	1
**bean**	µg/kg)	min	nd	<CCα	nd	nd	464.9	nd	nd	nd	nd	nd	63.4	nd	21.1	nd	nd	85.1	<CCα	nd	112.8
**stover**		max	nd	<CCα	nd	nd	1793.7	nd	nd	nd	nd	nd	255.1	nd	21.1	nd	nd	603.2	<CCα	nd	112.8
**(n=5)**		mean	nd	<CCα	nd	nd	1129.3	nd	nd	nd	nd	nd	159.3	nd	21.1	nd	nd	344.2	<CCα	nd	112.8
**Silages**	n (pos)		0	5	0	1	0	0	0	0	0	0	2	0	1	0	0	0	2	0	1
		min	nd	76.8	nd	138.6	nd	nd	nd	nd	nd	nd	15.5	nd	50.5	nd	nd	nd	14.4	nd	225.2
**(n=6)**	(µg/kg)	max	nd	1004.9	nd	138.6	nd	nd	nd	nd	nd	nd	52.8	nd	50.5	nd	nd	nd	17.4	nd	225.2
		mean	nd	491.2	nd	138.6	nd	nd	nd	nd	nd	nd	34.2	nd	50.5	nd	nd	nd	15.9	nd	225.2

LEGEND: ** semi-quantitative result, nd: not detected Nivalenol (NIV), deoxynivalenol (DON), 3- acetyl deoxynivalenol (3-ADON), 15- acetyl deoxynivalenol (15-ADONs), zearalenone (ZEN), aflatoxin G_2 (AFG_2), aflatoxin G_1 (AFG_1), aflatoxin B_2 (AFB_2), aflatoxin B_1 (AFB_1), diacetoxyscirpenol (DAS), alternariol (AOH), fumonisin FB_1 (FB_1,) fumonisin FB_2 (FB_2), fumonisin FB_3 (FB_3), ochratoxin A (OTA), alternariol monomethylether (AME), sterigmatocystin (STERIG), roquefortine C (ROQ-C) and enniatin B (ENN B).

Table S3. Overview of prevalence data of investigated mycotoxins in smallholder dairy feeds specifying individual farm means concentrations alongside overall contamination data (N=77).

FARM	Nivalenol	Deoxynivalenol	Neosolaniol	Fusarenon-X	3- Acetyl Deoxynivalenol	15- Acetyl Deoxynivalenol	Zearalenone	Aflatoxin G2	Aflatoxin G1	Aflatoxin B2	Aflatoxin B1	Diacetoxyscirpenol	Alternariol	Fumonisin FB1	Fumonisin FB2	Fumonisin B3	T-2 -Toxin	HT2-Toxin	Ochratoxin A	Alternariol Monomethyl Ether	Sterigmatocystin	Roquefortine C	Enniatin
1 n=2)	/	/	/	/	/	/	<u>1129.3</u>	/	/	/	/	/	127.6	/	10.6	/	/	/	/	344.2	/	/	/
2 (n=4)	/	/	/	/	/	/	/	/	/	/	/	/	232.0	/	/	/	/	/	/	/	34. 8	/	/
3 (n=2)	/	/	/	/	/	/	/	/	/	/	/	/	/	/	/	/	/	/	/	/	8.2	/	/
4 (n=2)	/	/	/	/	/	/	48.4	/	/	/	/	/	64.5	/	/	/	/	/	/	69.9	/	/	/
5 (n=1)	/	/	/	/	/	/	/	/	/	/	/	/	/	/	/	/	/	/	/	/	/	/	/
6 (n=3)	/	338.1	/	/	23.4	/	/	/	/	/	/	/	152.1	69.5	71.6	/	/	/	/	/	/	/	153.1
7 (n=4)	/	145.9	/	/	/	52.7	/	/	/	/	/	/	772.1	61.8	/	/	/	/	/	/	/	174.9	1762.2
8 (n=4)	/	243.0	/	/	/	/	/	/	/	/	/	/	162.1	78.1	/	/	/	/	/	/	22.4	/	/
9 (n=2)	/	453.4	/	/	4.2	97.5	146.9	/	/	/	/	/	/	179.0	122.5	/	/	/	/	/	3.40	/	/
10 (n=3)	/	333.1	/	/	29.0	59.7	/	/	/	/	/	/	10.2	120.2	98.7	/	/	/	/	/	/	/	107.0
11 (n=2)	/	/	/	/	/	/	/	/	/	/	/	/	42.1	/	/	/	/	/	/	/	/	/	/
12 (n=1)	/	559.8	/	/	/	235.5	/	/	/	/	/	/	/	392.0	315.1	/	/	/	/	/	/	/	130.7
13 (n=3)	/	255.4	/	/	18.1	52.1	/	/	/	/	/	/	/	161.7	56.2	/	/	/	/	/	4.8	/	6.9
14 (n=4)	/	256.8	/	/	/	/	/	/	/	/	/	/	213.6	/	6.4	/	/	/	/	/	1.2	/	/
15 (n=4)	/	46.4	/	/	/	/	/	2.78	<u>10.1</u>	2.8	<u>13.0</u>	/	109.4	/	8.03	/	/	/	46.9	/	16.7	/	/
16 (n=2)	/	487.5	/	/	/	44.4	/	/	/	/	/	/	16.1	/	/	/	/	/	/	/	4.7	/	10.3
17 (n=5)	/	179.0	/	/	3.5	10.6	/	/	/	/	/	/	52.9	/	/	/	/	/	3.9	/	16.2	10.9	143.6
18 (n=5)	/	234.2	/	/	/	/	/	/	/	/	/	/	36.1	/	10.1	/	/	/	/	17.7	3.0	/	45.0
19 (n=5)	/	403.8	/	/	5.1	24.8	/	/	/	0.4	/	/	54.3	93.9	83.4	/	/	/	9.9	/	8.5	/	18.5
20 (n=1)	/	258.0	/	/	13.6	57.7	/	/	/	/	/	/	89.1	/	/	/	/	/	/	/	9.00	/	/
21 (n=9)	4.1	63.6	/	/	/	16.0	/	/	/	/	/	/	50.8	/	/	/	/	/	/	/	5.1	/	864.6
22 (n=9)	/	583.3	/	/	43.2	141.1	223.7	/	/	/	/	0.4	112.0	43.7	22.2	/	/	/	/	/	11.6	/	249.3
[1]Overall Mean (μg/kg)	36.9	477.8	ND	ND	55.5	169.6	666.0	11.1	20.2	4.43	26.1	3.4	279.3	189.9	132.4	ND	ND	ND	85.6	229.2	25.8	377.2	1195.1
[1]Range (μg/kg)	36.9	76.8 – 2385.4	ND	ND	8.4 – 300.0	16.0 – 858.8	96.7 – 1793.7	11.1	17.2 – 23.1	2.2 – 6.8	21.9 – 30.2	3.4	15.5 – 3088.2	39.3 – 485.2	21.1 – 416.9	ND	ND	ND	19.4 – 187.9	85.1 – 603.2	4.8 – 139.1	54.4 – 699.9	20.6 – 14230.4
[1]N (%) pos	1 (1.3)	38 (48.7)	ND	ND	12 (15.4)	16 (20.5)	7 (8.9)	1 (1.3)	2 (2.6)	3 (3.9)	2 (2.6)	1 (1.3)	33 (42.9)	17 (21.8)	15 (19.2)	ND	ND	ND	3 (3.9)	4 (5.1)	25 (32.1)	2 (2.6)	22 (28.2)
[1]N (%) > legislated limits		1 (1.3) EU					2 (2.6) EU/ SA				2 (2.6) SA												

LEGEND: [1]Overall data with values above CCα (positive samples); **CCα**: decision limit; /: none quantified result (ND-not detected or <CCα -decision limit); **<u>Underlined farm mean</u>** values exceed legislated limits apart from farm 4/T-2 toxin as this value was estimated; [1]**N (%) pos**: number and percentage of positive samples. **N (%) > legislated limits**: number and percentage of positive samples above legislated limits (DON: EU 2 000 μg/kg; ZEN: SA/EU 500 μg/kg; AFB1: SA 5 μg/kg/EU: 20 μg/kg).

Table S4. Mycotoxin co-occurrence combinations found in study.

Number of coexisting mycotoxins	Number of combinations	Types of combinations (a)	
7	1	AFG2, AFG1, AFB2, AFB1, FB2, AOH, STERIG	
6	6	DON, 3ADON, 15ADON, FB1 FB2, AOH<br>DON, 3ADON, 15ADON, FB1, FB2, ENN B<br>DON, 3ADON, ZEN, AOH, STERIG, ENN B	DON, 15ADON, AFB2, AFB1, AOH, STERIG<br>DON, FB1, FB2, AOH, STERIG, ENN B<br>DON, AFG1, AFB2, AFB1, OTA, STERIG
5	12	DON, 3ADON, 15ADON, FB1, FB2<br>DON, 3ADON, 15ADON, FB1, DAS<br>DON, 3ADON, 15ADON, FB1, STERIG<br>DON, 3ADON, 15ADON, OTA, ENN B<br>DON, 3ADON, 15ADON, OTA, STERIG<br>DON, 3ADON, 15ADON, AOH, STERIG	DON, 15ADON, FB1, FB2, ENN B<br>DON, 15ADON, FB1, FB2, ZEN<br>DON, 3ADON, FB1, FB2, ENN B<br>DON, 3ADON, FB1, FB2, STERIG<br>DON, AOH, STERIG, ROQ-C, ENN B<br>DON, FB1, FB2, ZEN, ENN B
4	5	DON, 15ADON, ZEN, STERIG<br>DON, 3ADON, FB1, FB2<br>DON, FB1, FB2, ENN B	DON, FB1, AOH, STERIG<br>DON, AOH, STERIG, ENN B
3	12	DON, AOH, STERIG<br>DON, AOH, ENN B (2)<br>DON, FB2, AOH<br>DON, 15ADON, STERIG<br>DON, 15ADON, ENN B<br>DON, 15ADON, FB1	ZEN, AOH, AME (2)<br>ZEN, FB2, AME<br>FB2, AOH, STERIG<br>NIV, AOH, ENN B<br>AOH, STERIG, ENN B<br>AOH, STERIG, AME
2	8	DON, FB1 (2)<br>DON, AOH (3)<br>DON, 15ADON<br>DON, ENN B	AOH, STERIG (2)<br>AOH, ENN B<br>ROQ-C, ENN B<br>STERIG, ENN B (2)
1	5	DON<br>T2<br>STERIG (3)	AOH (8)<br>ENN B (2)
0	11	-	

Notes: (n) number of multiple occurrences of combination. Nivalenol (NIV), deoxynivalenol (DON), 3- acetyl deoxynivalenol (3-ADON), 15- acetyl deoxynivalenol (15-ADONs), zearalenone (ZEN), aflatoxin G2 (AFG2), aflatoxin G1 (AFG1), aflatoxin B2 (AFB2), aflatoxin B1 (AFB1), diacetoxyscirpenol (DAS), alternariol (AOH), fumonisin FB1 (FB1,) fumonisin FB2 (FB2), ochratoxin A (OTA), alternariol monomethyl ether (AME), sterigmatocystin (STERIG), roquefortine C (ROQ-C), enniatin B (ENN B).

Table S5. LC Gradient program employed.

**Time (minutes)**	**0**	**7**	**11**	**13**	**14**	**14.1**	**17.6**	**18.6**	**19.8**	**19.9**	**22.4**	**23.4**	**25**	**26**	**28**
Mobile phase A (%)	95	35	25	1	1	95	35	25	1	95	35	25	1	95	95
Mobile phase B (%)	5	65	75	99	99	5	65	75	99	5	65	75	99	5	5

Table S6. MS/MS parameters for determination of 23 mycotoxins and 2 internal standards.

**Mycotoxin**	**Precursor ion (m/z)**	**Fragment ions (m/z)**	**Collision energy (eV)**	**Cone voltage (V)**	**Expected retention time (min)**
Aflatoxin B1	313.0	285.1*<br>241.2	24.0<br>36.0	51.0<br>51.0	7.9
Aflatoxin B2	315.0	287.2*<br>259.2	27.0<br>30.0	51.0<br>51.0	7.6
Aflatoxin G1	329.0	243.0*<br>311.2	25.0<br>20.0	44.0<br>44.0	7.2
Aflatoxin G2	331.0	313.1*<br>245.2	25.0<br>30.0	53.0<br>53.0	6.9
Ochratoxin A	403.9	239.0*<br>358.2	22.0<br>14.0	24.0<br>24.0	11.3
Deoxynivalenol	297.1	249.2*<br>231.2	10.0<br>15.0	26.0<br>26.0	4.3
Zearalenone	319.1	187.2*<br>203.0	19.0<br>20.0	27.0<br>27.0	11.7
Fumonisin B1	722.1	352.4*<br>704.4	36.0<br>29.0	56.0<br>56.0	9.4
Fumonisin B2	706.1	336.5*<br>688.5	40.0<br>29.0	61.0<br>61.0	10.6
Fumonisin B3	706.1	336.5*<br>688.5	37.0<br>31.0	54.0<br>54.0	12.0
T-2 toxin	489.1	245.1*<br>327.0	26.0<br>26.0	26.0<br>26.0	10.2
HT-2 toxin	447.1	345.3*<br>285.1	21.0<br>23.0	30.0<br>30.0	9.2
Nivalenol	313.1	125.0*<br>205.0	13.0<br>12.0	26.0<br>26.0	3.2
3- Acetyl deoxynivalenol	339.0	231.2*<br>203.2	13.0<br>12.0	23.0<br>23.0	6.5
15- Acetyl deoxynivalenol	339.0	137.1*<br>321.2	10.0<br>10.0	26.0<br>26.0	6.5
Diacetoxyscirpenol	384.2	307.1*<br>247	12.0<br>14.0	21.0<br>21.0	7.9
Fusarenon-X	355.0	174.9*<br>137.0	20.0<br>25.0	18.0<br>18.0	5.2
Neosolaniol	400.0	305.3*<br>185.0	12.0<br>19.0	26.0<br>26.0	5.3
Alternariol	258.9	185.1*<br>213.1	30.0<br>26.0	40.0<br>40.0	9.9
Alternariol Methyl Ether	272.9	258.2*	26.0	57.0	12.7

		199.3	30.0	57.0	
Roquefortine C	390.0	193.2*	26.0	40.0	8.9
		322.2	21.0	40.0	
Sterigmatocystin	325.0	310.2*	25.0	47.0	12.2
		281.1	36.0	47.0	
Enniatin B	622.0	336.3*	49.0	58.0	13.0
		549.4	46.0	58.0	
Zearalanone (IS)	321.0	303.3*	13.0	26.0	11.3
		189.2	19.0	26.0	
De-epoxydeoxynivalenol (IS)	281.0	109.1*	19.0	26.0	5.5
		137	15.0	26.0	

* most abundant ion.

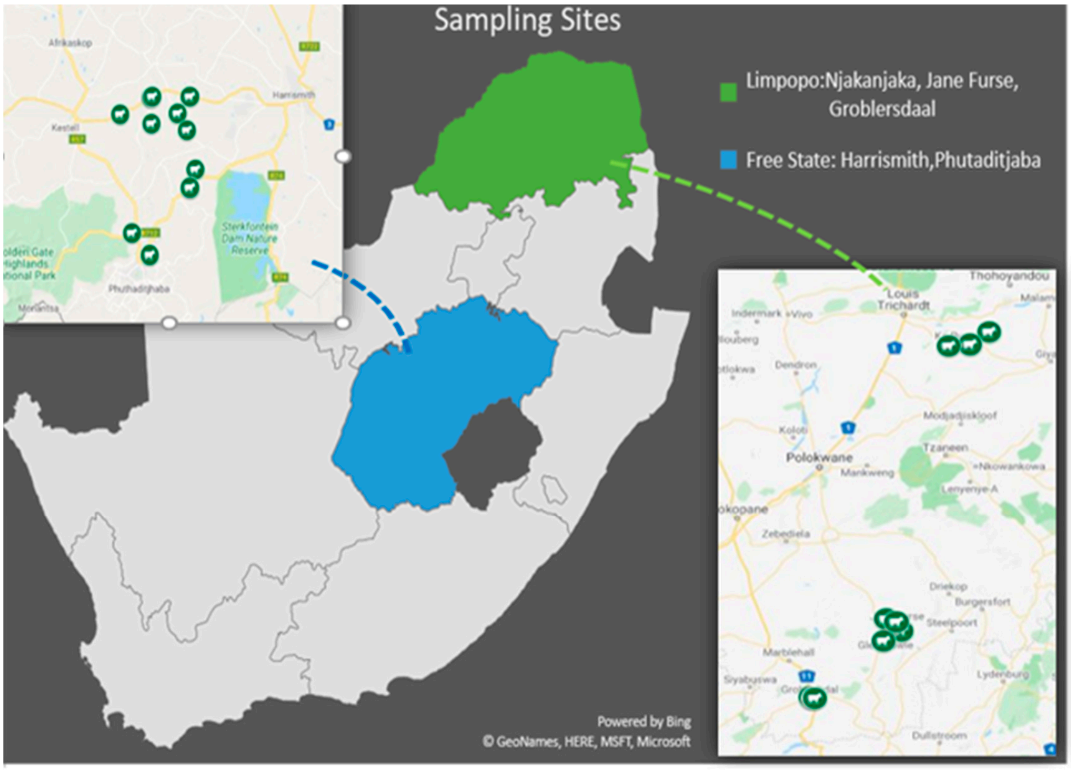

Figure S1. Map of South Africa showing towns and smallholder farming areas of interest to this study.
